# Untargeted CUT&Tag reads are enriched at accessible chromatin and restrict identification of potential G4-forming sequences in G4-targeted CUT&Tag experiments

**DOI:** 10.1093/nar/gkaf678

**Published:** 2025-07-19

**Authors:** Matthew D Thompson, Alicia K Byrd

**Affiliations:** Department of Biochemistry and Molecular Biology, University of Arkansas for Medical Sciences, Little Rock, AR 72205, United States; Department of Biochemistry and Molecular Biology, University of Arkansas for Medical Sciences, Little Rock, AR 72205, United States; Winthrop P. Rockefeller Cancer Institute, Little Rock, AR 72205, United States

## Abstract

G-quadruplex DNA structures (G4s) form within single-stranded DNA in nucleosome-free chromatin. G4s modulate gene expression and genomic stability, so high-throughput, genome-wide mapping of G4s has generated strong research interest and methodological innovation. Recently, the Cleavage Under Targets and Tagmentation (CUT&Tag) method has been adapted to map G4s using an antibody, a nanobody, and G4-binding small molecules to target Tn5 tagmentation to G4s. These novel methods have generated high-resolution maps of G4s, but we have observed a strong colocalization between untargeted and G4-targeted CUT&Tag signal enrichment, leading us to wonder whether this colocalized signal enrichment would impact G4 mapping using these methods. We observed that the genome-wide signal distribution of untargeted CUT&Tag libraries was highly correlated with that of both cell-line-matched ATAC-seq libraries and cell-line-matched G4-mapping CUT&Tag libraries. When peaks were called from G4-mapping CUT&Tag libraries using the SEACR algorithm with inclusion of the respective matched untargeted CUT&Tag libraries, certain peaks at potential G4-forming sequences were excluded, slightly enhancing precision with which G4s are mapped while limiting recall of potential G4s. Consequently, we recommend that care be exercised when interpreting G4-targeted CUT&Tag experiments unless untargeted tagmentation is taken into account or minimized through protocol optimization.

## Graphical Abstract

**Graphical Abstract:**
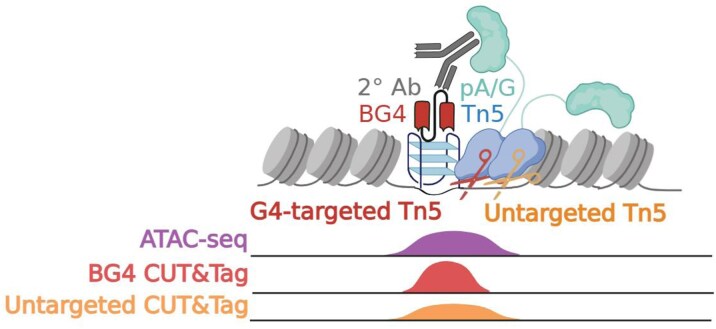

## Introduction

Formation of non-B DNA secondary structures is both a driver and a consequence of myriad biological processes (reviewed in [1]). G-quadruplex DNA structures (G4s) are present throughout regulatory regions of the human genome [2] and influence a diverse array of cellular processes such as gene expression [3–5], DNA replication progression [6–8], and DNA replication origin firing [9, 10]. G4s are significantly enriched in cancerous tissues relative to normal tissues [11], and due to their prevalence in telomeres and promoters of proto-oncogenes, they are therapeutic targets with two G4 ligands currently in clinical trials [12, 13]. G4s are formed by runs of multiple guanine bases that interact via Hoogsteen base pairing to form planar tetrads [14] that stack into a folded G4 structure [15]. A related DNA secondary structure, the i-motif, can also be formed at C-rich sequences through intercalated Hoogsteen base pairing of hemiprotonated cytosines (reviewed in [16, 17]). G4s can be bound and/or resolved by endogenous proteins such as the G4-binding Sp1 transcription factor [3] and helicase DHX36/RHAU [18, 19], and i-motifs can be bound and/or resolved by proteins such as hnRNP K [20] and hnRNP LL [21, 22]. Binding of proteins to these DNA secondary structures can modulate processes such as transcription [4, 5], and resolution of these secondary structures is crucial to safeguarding genomic stability [23, 24] (reviewed in [25]). Additionally, there exist ongoing efforts to selectively stabilize/destabilize G4s and i-motifs *in cellulo* with chemical ligands to modulate translationally relevant biological processes (reviewed in [26–28]). Consequently, efforts to identify and map folded G4s and i-motifs throughout the human genome have generated significant research interest (reviewed in [29] for G4s; see [30, 31] for i-motifs).

Initial G4 mapping efforts relied on algorithmic prediction (reviewed in [32]) and next-generation sequencing methods [33, 34] to identify sequences with the potential to form G4s *in vitro*. Subsequent methodological innovations have enabled capture and next-generation sequencing of G4-forming loci. These can be broadly classified by DNA fragmentation method. The first class of methods utilizes G4-targeted immunoprecipitation of sonicated chromatin followed by sequencing (i.e. ChIP-seq). Immunoprecipitation of G4s has been achieved using multiple proteins such as the single-chain variable fragment antibody BG4 [35], the nanobody SG4 [36], and the G4P polypeptide probe [37] constructed from tandem-linked G4-binding helices from the RHAU/DHX36 helicase [18, 19]. Additionally, use of the iMab antibody [38–40] has recently allowed for mapping of i-motifs using ChIP-seq [31]. The second class consists of the G4Access mapping method, which utilizes controlled enzymatic digestion of accessible chromatin with micrococcal nuclease (MNase) to map G4s through the preference of MNase for cutting before G-rich sequences and the resistance of G4-forming sequences to exonuclease cleavage [41]. The third class of G4 mapping methods involves use of the Tn5 transposase to fragment and tag DNA in the vicinity of G4s with sequencing adapters (i.e. “tagmentation”), following antibody-mediated targeting of a Protein A-Protein G-Tn5 fusion protein to G4s or i-motifs. These methods include BG4 CUT&Tag [5, 42–44], SG4 CUT&Tag [5], iMab CUT&Tag [30], and Chem-map [5, 45], which targets the Tn5 fusion protein to G4s through Protein A/G interactions with anti-biotin antibodies that recognize the biotinylated G4-stabilizing small molecules pyridostatin (PDS) or PhenDC3. These recently developed Tn5-based methods offer improvements over the ChIP-seq-based G4 mapping methods with higher signal-to-noise ratios, the possibility of native (i.e. unfixed) conditions, fragmentation of DNA within the cellular context, and lower requirements for the amount of input material [46, 47], thus allowing for G4 mapping in samples with limiting cell populations.

To identify these DNA secondary structures within the next-generation sequencing libraries produced by these techniques, multiple algorithms such as Model-based Analysis of ChIP-seq (MACS; [48]) and Sparse Enrichment Analysis for CUT&RUN (SEACR; [49]) are employed, with the MACS algorithm employed for high-coverage, low signal-to-noise ChIP-seq-based libraries and the SEACR algorithm employed for low-coverage (i.e. sparse), high signal-to-noise CUT&Tag and Cleavage Under Targets and Release Using Nuclease (CUT&RUN) libraries. For ChIP-seq-based assays and G4Access, an “input” sample composed of sonicated whole genomic chromatin is saved prior to immunoprecipitation or enzymatic digest, as the remaining pieces of this fragmented DNA bound by the target of interest are then captured by immunoprecipitation. The negative control input library is then sequenced alongside the immunoprecipitated chromatin, providing an estimate of nonspecific DNA amplification and sequencing, which could bias interpretation of the sequencing results from the immunoprecipitated DNA. The genome-wide background from this input sample is then utilized by the MACS algorithm as the expected value of genomic background reads in a Poisson distribution to allow for identification of genomic loci with statistically significant enrichment of the immunoprecipitated DNA compared to the local genomic background [48]. In contrast, the CUT&Tag-based G4 mapping assays are typically performed alongside a matched untargeted CUT&Tag reaction to measure genomic background. However, these untargeted CUT&Tag controls are likely to contain signal produced by untargeted Tn5 transposase tagmentation at nucleosome-depleted, open chromatin [46, 47, 50, 51]. This is unsurprising, given that untargeted Tn5 cleavage is typically used for mapping of open chromatin in the assay for transposase-accessible chromatin with sequencing (ATAC-seq) [52]. Noting this effect, the original authors of the CUT&Tag method used a read-count discrimination method to distinguish between targeted CUT&Tag cleavage and untargeted cleavage, with the assumption that targeted CUT&Tag events would occur with greater frequency at the targeted loci, when compared to untargeted cleavage at accessible chromatin [46]. This read count discrimination approach has been validated for use with histone post-translational modifications and the CTCF transcription factor [46], but a similar analysis to validate this prior approach has not been performed for Tn5-based G4 mapping methods. Consequently, we seek in this manuscript to investigate the genome-wide enrichment patterns of untargeted CUT&Tag libraries and quantify whether the use or omission of untargeted CUT&Tag libraries influences identification of G4-forming loci in G4-targeted CUT&Tag libraries.

Given that G4s require an open chromatin environment to form, we hypothesized that untargeted Tn5-catalyzed tagmentation at open chromatin during CUT&Tag could generate local signal enrichment in the vicinity of G4s and could impair detection of G4s due to overlap between the targeted G4-mapping CUT&Tag signal and the untargeted CUT&Tag signal. To test this, we compiled untargeted CUT&Tag libraries and assessed the local sequence and chromatin environment at sites of signal enrichment in the untargeted CUT&Tag libraries. We additionally compared enrichment of G4-targeted CUT&Tag libraries from BG4 CUT&Tag, SG4 CUT&Tag, and Chem-map over matched untargeted CUT&Tag libraries at mapped G4s to assess the capability of these methods to specify the presence or absence of G4s. Although the overall amplitude of G4-targeted CUT&Tag signal increases at G4s and shares high correlation with signal produced by other orthogonal G4-mapping methods, we observed that the enrichment of G4-targeted CUT&Tag signal above untargeted CUT&Tag signals in matched controls is not always significantly increased at these sites. This makes a differential read count method for mapping G4s intractable for certain G4-mapping libraries and limits recall of G4s in G4-targeted CUT&Tag libraries.

## Materials and methods

### Data acquisition and processing

Untargeted CUT&Tag libraries in the Gene Expression Omnibus (GEO) [53] were queried using the following: (CUT AND Tag AND “homo sapiens”) AND “.bw” AND ((“gse”[Filter] OR “gds”[Filter] OR “gsm”[Filter]) AND “Homo sapiens”[Organism]). Reads for each library used were downloaded from the sequence read archive (SRA) [54] as .fastq files using prefetch and fasterq-dump with default settings (https://github.com/ncbi/sra-tools, v3.1.1) using the SRA accessions indicated (Supplementary Tables S1, S4, S7, and S9). Raw reads from paired-end libraries were processed using the Nextflow [55, 56] v23.10.0 cutandrun pipeline, v3.2.1 [57], and raw reads from single-end libraries (ChIP-seq) were similarly processed using the Nextflow v23.10.0 chipseq pipeline, v2.0.0 [58]. Briefly, reads were filtered with fastqc [59], trimmed to remove adapters with Trim Galore [60] (i.e. a wrapper for Cutadapt [61]), aligned and paired with Bowtie2 [62] to the GRCh38 assembly of the human genome, and deduplicated with Picard [63] unless otherwise indicated. Mapped reads that overlapped the ENCODE Hg38 blacklist, v2 [64], were removed prior to subsequent analyses, and reads from technical replicates were pooled following alignment.

### Peak calling

Peaks from CUT&Tag libraries were called using SEACR v1.3 [49]. Briefly, deduplicated alignments were sorted with SAMtools *sort* [65], converted to paired-end bed files with bedtools *bamtobed -bedpe* [66], and converted to coverage files using bedtools *genomecov* [66] across the hg38 assembly. For untargeted CUT&Tag libraries, SEACR was utilized with the indicated threshold in stringent mode without a negative control. When a control library was utilized for calling peaks in targeted CUT&Tag libraries, SEACR was used with in-algorithm normalization (i.e. *norm*) with the indicated stringency setting. For ChIP-seq and G4Access libraries, MACS3 v3.0.2 *callpeak* [48] was run with local lambda estimation from input controls with a q-value cutoff of 0.05. For ATAC-seq libraries, MACS3 v3.0.2 *callpeak* was run without a negative control and without local lambda estimation with a q-value cutoff of 0.05. Any peaks called from nonstandard chromosomes within the GRCh38 assembly were omitted.

### Library complexity normalization

For Tn5-derived libraries, library complexity was estimated using ATACseqQC [67] in R. Briefly, duplication rates were calculated by passing in non-deduplicated alignments to *readsDupFreq*, followed by utilization of this output in *estimateLibComplexity*. The estimated number of unique paired-end reads was then divided by the total number of paired-end reads to calculate the proportion of unique reads per library. Downsampling of non-deduplicated libraries was performed using SAMtools *view* [65] with a random seed of 1 and a threshold to downsample each library to approximate the limiting number of estimated unique paired-end reads in the lowest complexity library, prior to subsequent deduplication. The threshold was derived by dividing the desired number of unique reads by the total estimated number of unique reads for each library.

### Read visualization

Read counts were normalized by read depth using deeptools *bamCoverage* [68] with counts per million reads (CPM) normalization with a bin size of 50, an effective genome size of 2 913 022 398, and paired-end read centering (i.e. *--centerReads*) for paired-end libraries. Normalized read counts were plotted on a linear scale according to the indicated axis limits using IGV [69].

### Library read count correlation

Libraries were compared using DiffBind [70, 71] in R. Briefly, counts of any reads mapping within called peaks present in any provided library were calculated from the adapter-trimmed, quality-filtered, and deduplicated (or complexity-normalized) libraries output by the nextflow pipeline(s) by *dba.count* with *minOverlap = 0*, *bUseSummarizeOverlaps = FALSE*, *minCount = 0*, *bSubControl = FALSE*, *filter = 0*, *filterFun = sum*, and *mapQCth = 0*. Fraction of reads in peaks (FRiP) scores were derived from this output. Read counts were then normalized by *dba.normalize* with *background = TRUE* with a computed library size of *DBA_LIBSIZE_BACKGROUND* and *normalize = DBA_NORM_NATIVE* (i.e. using DESeq2 [72]) as recommended for libraries lacking spike-ins or parallel factors [71].

Principle component analysis (PCA) plots were generated using *dba.plotPCA* with default settings on the libraries following read count normalization. Read count correlation heatmaps were generated using DiffBind *plotHeatmap* with default settings on the libraries following read count normalization.

### Peak overlap analysis

Genomic intervals present in peaks between more than two libraries were identified using bedtools *multiinter*[66] with default settings, followed by selection of intervals present in the desired number of libraries and merging of directly adjacent intervals with bedtools *merge*[66]. Precision/recall and F1 scores of peaks from target libraries against peaks from reference libraries were calculated using EpiCompare *precision_recall* [73] in R. Jaccard statistics were calculated using bedtools *jaccard* [66] with default settings for target libraries and shuffled controls. Fisher’s exact test was performed using bedtools *fisher* [66] with default settings. Shuffled control peaks were generated using bedtools *shuffle* [66] with the target library as input, with the exclusion of ENCODE blacklist regions, with the exclusion of nonstandard chromosomes from the available shuffle genome coordinates, and with the prevention of overlapping intervals following shuffling.

### Epigenetic annotation and motif discovery

Peaks in target libraries were annotated using ChIPseeker *annotatePeak* [74] using the GenomicFeatures hg38 annotation set [75], followed by plotting using ChIPseeker *plotAnnoPie* [74]. Peaks were also annotated using HOMER *annotatePeaks* [76] with default settings using the hg38 assembly. Motifs were found using HOMER *findMotifsGenome* [76] with default settings.

### Differential enrichment analyses

Differential enrichment analyses were performed using DiffBind [70, 71]. Briefly, deduplicated or complexity-normalized libraries of G4-mapping CUT&Tag or matched untargeted CUT&Tag controls were normalized as earlier (see the “Library read count correlation” section) across any peak of enrichment previously called for each library (0.01 threshold). Differential enrichment analysis was then performed using DiffBind *dba.analyze* [70, 71] with default settings for DEseq2-mediated relative-log expression normalization, locally estimated scatterplot smoothing (LOESS) curve fitting, and analysis [72]. Any peak of enrichment across all samples with a significant (false discovery rate [FDR] < 0.05) increase in normalized read counts between the G4-targeted CUT&Tag library replicates and the matched untargeted CUT&Tag libraries was highlighted in magenta after plotting with DiffBind *plotMA* [70, 71] with default settings.

## Results

### Low-complexity untargeted CUT&Tag samples have a shared pattern of genome-wide enrichment

**Figure 1.**
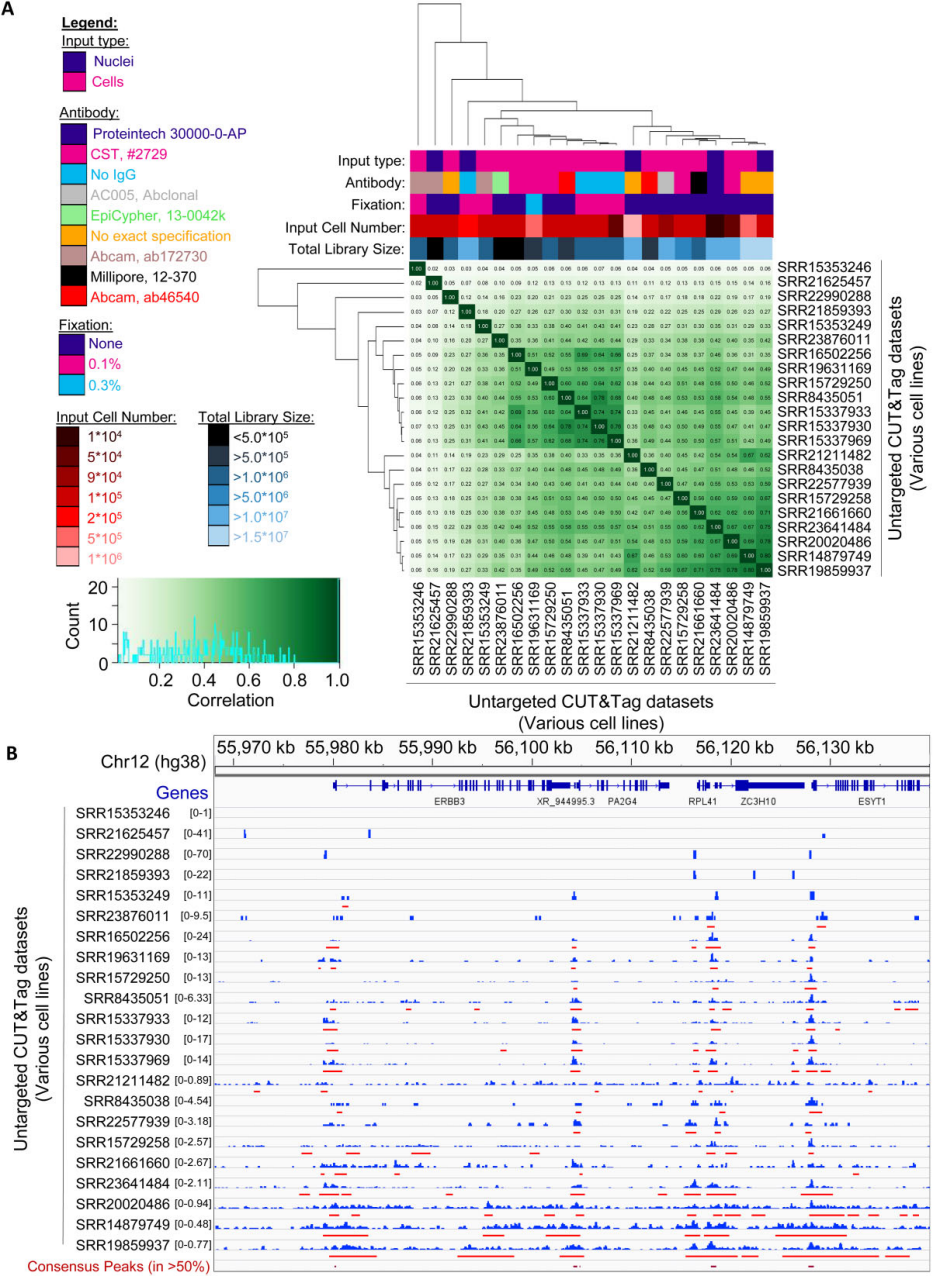
Untargeted CUT&Tag libraries from multiple cell lines exhibit a modest correlation of genome-wide signal enrichment. (**A**) Clustered heatmap of Pearson’s correlation coefficients of normalized read counts from deduplicated untargeted CUT&Tag libraries across any site of signal enrichment of each sample. Associated sample preparation and sequencing depth features are indicated by color, according to provided scale (left). The correlation value distribution histogram is plotted within the Pearson’s correlation coefficient color scale (bottom left). (**B**) CPM-normalized read counts (blue) of deduplicated untargeted CUT&Tag libraries, ordered vertically as in panel (A) at a representative locus on chromosome 12. Read counts are plotted on the indicated axes to highlight the maximum signal enrichment for each sample at this representative locus. SEACR-called peaks of signal enrichment (red) are plotted for each library, alongside a set of consensus peaks (maroon; *n* = 2857), derived from genomic intervals present in at least 50% of the libraries.

We acquired untargeted CUT&Tag libraries from publicly available data (Supplementary Table S1) to assess whether these data display any pattern of genome-wide enrichment. We selected libraries originating from a variety of cell lines and produced by multiple different labs in an attempt to capture any library- and cell line-agnostic signal enrichment in untargeted CUT&Tag libraries. Raw reads were reprocessed using nextflow cutandrun [57] with deduplication, and peaks were called using SEACR [49] using a threshold to only retain the top 5% of signal blocks as peaks (i.e. a threshold of 0.05) (Supplementary Table S2). Assessment of correlation between the CPM-normalized read counts of the untargeted CUT&Tag libraries across the loci called as peaks revealed a broad range of correlation strengths between the read count distributions of each library (Fig. 1A). However, when read count distributions and peaks of local enrichment were examined at a representative locus, we observed a spatially correlated pattern of signal enrichment (Fig. 1B; plotted to emphasize signal enrichment within each sample). Of note, when these data were plotted on a uniform amplitude scale, the local untargeted CUT&Tag signal enrichment was difficult to visualize due to strong variations in signal amplitudes (Supplementary Fig. S1A) and signal-to-noise ratios across the datasets (Supplementary Table S2). We also observed exceptions to the observed spatial correlation, such as libraries SRR15353246 and SRR2165457, possibly attributable to very low library complexity and/or very low sequencing depth (Supplementary Table S2). Despite these variations in signal amplitude and exceptions, we were able to derive a set of consensus peaks (Fig. 1B, bottom track) to mark loci with signal enrichment in at least half of the examined datasets.

Next, we examined sample preparation factors that could contribute to the differences and similarities in genome-wide signal, such as use of non-targeting antibody, antibody sources, and formaldehyde fixation (Supplementary Table S3). Most of the factors examined did not strongly correlate with major differences in read count distributions when visualized using PCA (Supplementary Fig. S2A–E). The first two principle components only accounted for around 50% of the observed variation, indicating that the examined datasets are diverse with respect to genome-wide read enrichment of untargeted CUT&Tag reads, despite the observed spatially correlated peaks of signal enrichment. Libraries that were spatially distinct in the PCA visualization (labeled by accession in Supplementary Fig. S2A) still had notable read count correlation (Fig. 1A) but had lower normalized signal intensity when compared to other samples at a representative locus on chromosome 12 (Fig. 1B). A cluster of similar libraries was observed when fixation status of libraries was examined (Supplementary Fig. S2C), suggesting that fixation prior to untargeted CUT&Tag may reduce variation in read distributions. When libraries were compared by the total number of reads prior to deduplication (i.e. sequencing depth), we observed an apparent relationship between the spatial distributions of (i.e. similarity between) each library and the total sequencing depth (Supplementary Fig. S2F). A similar apparent relationship was also observed between the total number of unique reads in each library following deduplication (i.e. library complexity) and the spatial distribution of each library (Supplementary Fig. S2G). This suggested to us that a relationship between library complexity and observed genome-wide signal enrichment in untargeted CUT&Tag libraries might exist.

To examine this in a more quantitative manner, we examined the set of consensus peaks (intervals present in at least 50% of the libraries) from the untargeted CUT&Tag libraries (Fig. 1B, bottom track) and quantified the fraction of reads present within these peaks (i.e. FRiP score) as a way to measure the signal-to-noise ratio of each library relative to each other library (Supplementary Table S2). When the correlation between the signal-to-noise ratio and library complexity was quantified, no significant (*r*^2^= 0.17, *P*= 0.057) correlation was observed (Supplementary Fig. S2H). This indicates that the variation observed in the observed read distributions in the untargeted CUT&Tag libraries is not strongly attributable to any of the compared sample preparation factors besides fixation. Although these examined sample preparation factors do not appear to strongly correlate with library similarity (Supplementary Fig. S2A–E), it is conceivable that other unknown sample preparation factors such as cell permeability (determined by cell type, permeabilization buffer components, etc.) and/or Tn5 transposase source, purity, activity, and concentration relative to available genomic DNA content during tagmentation could contribute strongly to these library complexity differences. Polymerase chain reaction (PCR) amplification biases could also contribute to the observed signal and library complexity in non-deduplicated libraries; indeed, the low amount of DNA produced in the untargeted tagmentation reactions when combined with sequencing depth would be expected to influence the overall PCR duplication rate of the non-deduplicated libraries [77]. However, PCR duplication rates are not a contributing factor in the enrichment patterns observed here as the signal enrichment in the untargeted CUT&Tag libraries is observed after deduplication (Fig. 1). Additionally, greater sequencing depth can produce greater library complexity (i.e. a greater number of unique molecules), with the magnitude of the effect being impacted by the degree of saturation to which the library was sequenced [77]. This sequencing depth effect may impact the library complexity of the untargeted CUT&Tag libraries, given that most of the untargeted libraries are sequenced with much lower depth when compared to their matched targeted CUT&Tag libraries. These results reveal that a high amount of variation can be present in the read count distributions of untargeted CUT&Tag libraries with respect to signal amplitude and signal-to-noise ratios across different cell lines, but the observed spatial correlations of signal enrichment between these libraries remain notable.

**Figure 2.**
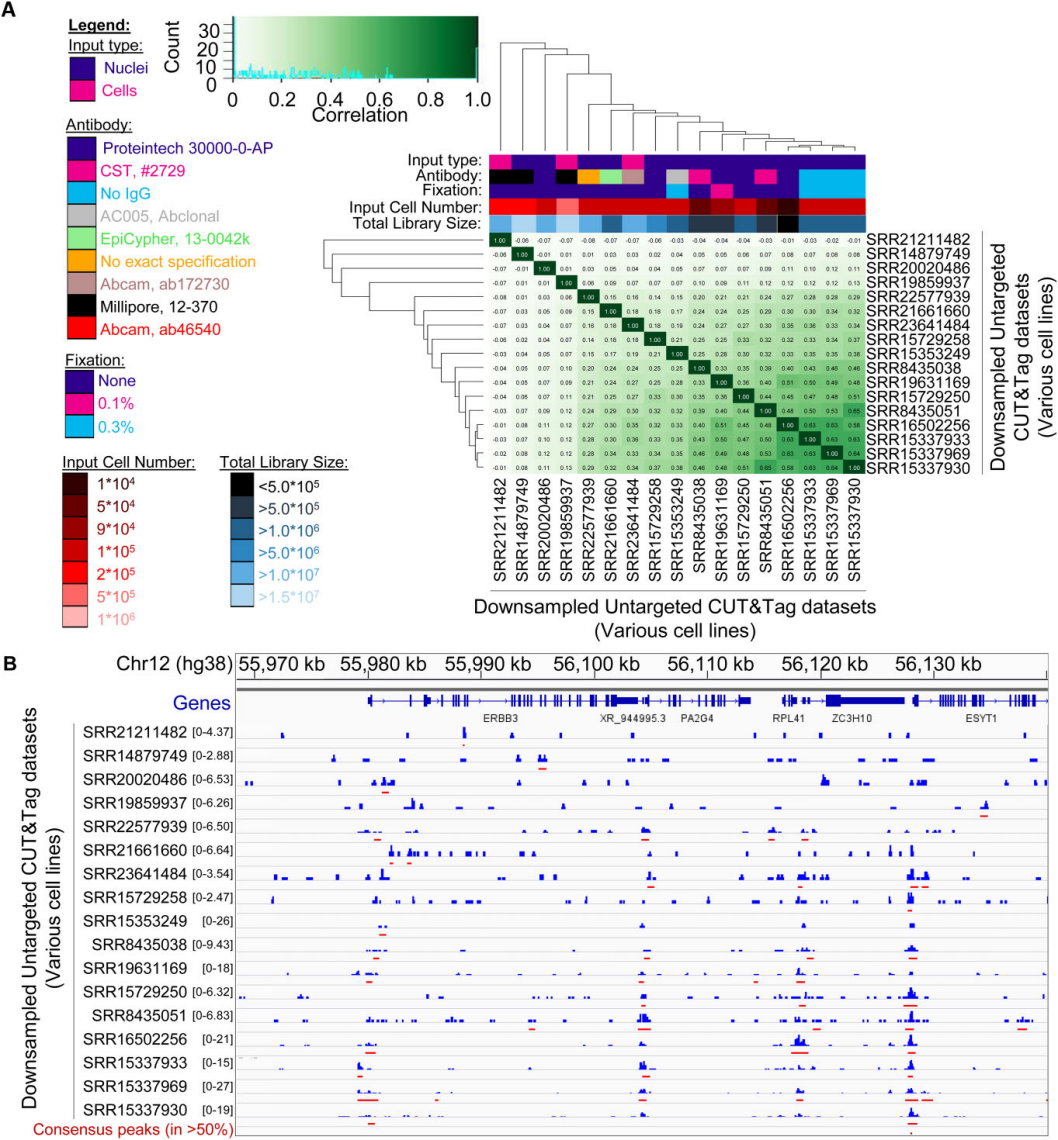
Genome-wide correlation of untargeted CUT&Tag libraries remains following library complexity normalization. (**A**) Clustered heatmap of Pearson’s correlation coefficients of normalized read counts from complexity-normalized (i.e. downsampled and deduplicated) untargeted CUT&Tag libraries across any site of signal enrichment of each sample. Associated sample preparation and sequencing depth features are indicated by color, according to provided scale (left). The correlation value distribution histogram is plotted within the Pearson’s correlation coefficient color scale (top left). (**B**) CPM-normalized read counts (blue) of the complexity-normalized untargeted CUT&Tag libraries, ordered vertically as in panel (A) at a representative locus on chromosome 12. Read counts are plotted on a linear scale on the indicated axes to highlight the maximum signal enrichment for each sample at this representative locus. SEACR-called peaks of signal enrichment (red) are plotted for each library, alongside a set of consensus peaks (maroon; *n* = 1032), derived from genomic intervals present in at least 50% of the libraries.

Noting the variations in signal amplitudes and sequencing depth across the untargeted CUT&Tag libraries (Supplementary Figs S1 and S2), we hypothesized that normalizing the complexity of each library through downsampling and deduplication could account for any differences in overall tagmentation distribution that had been skewed by these variations between the untargeted CUT&Tag libraries. As Tn5 from different sources (both commercially available and in-house-purified) vary in purity and presence of *Escherichia coli* DNA, we opted against using the previously recommended method [78] of quantifying reads from contaminating *E. coli* DNA in each library as a relative library size control. We instead turned to a method of library complexity normalization recommended for properly assessing differential enrichment of read counts between ATAC-seq libraries [79]. For this, the number of unique molecules in each library was estimated (Supplementary Table S2) and was used to downsample non-deduplicated libraries to approximate the same expected number of unique molecules, followed by deduplication. This normalization works through the assumption that increased enrichment at a genomic locus should be reflected in the number of unique molecules (i.e. unique tagmentation events) observed at a locus. Thus, when the total number of unique molecules is normalized between each library under comparison, enrichment at a given locus in a certain library should be reflected as an increased read count relative to other libraries [79]. To ensure sufficient genome-wide coverage of reads from the already sparse CUT&Tag libraries after downsampling, the untargeted CUT&Tag libraries with <300 000 unique reads were excluded from further analysis, and the remaining libraries were downsampled to an estimated unique molecule count approximating that of SRR15353249 prior to deduplication (Supplementary Table S2). Following this complexity normalization step, the majority of the downsampled libraries retained a modest correlation (Fig. 2A; Supplementary Fig. S1B), indicating that accounting for differences in library complexity does not fully remove the correlation between untargeted CUT&Tag libraries; we interpret this remaining correlation as a measure of similar genome-wide enrichment across the correlated libraries. However, the libraries with the highest library complexity lost any significant pattern of genome-wide enrichment following downsampling (Fig. 2A and B). Additionally, when the impact of sample preparation factors (Supplementary Table S3) was again examined using PCA on the downsampled libraries (Supplementary Fig. S3A–F), the libraries with the highest sequencing depth had the greatest dissimilarity from the other libraries examined (Supplementary Fig. S3F). However, no correlation between the signal-to-noise ratio at the set of remaining consensus peaks (intervals present in at least 50% of libraries; Fig. 2B, bottom track) and the total number of unique reads was observed (Supplementary Fig. S3G). We interpret these results to indicate that untargeted CUT&Tag libraries generated with sufficient complexity (i.e. a high number of unique reads) may not exhibit a unique genome-wide enrichment pattern, but that other lower complexity untargeted CUT&Tag libraries retain a weak, yet reproducible pattern of genome-wide enrichment across multiple cell lines even after complexity normalization.

### Low-complexity untargeted CUT&Tag libraries are enriched at Tn5-accessible regulatory regions

**Figure 3.**
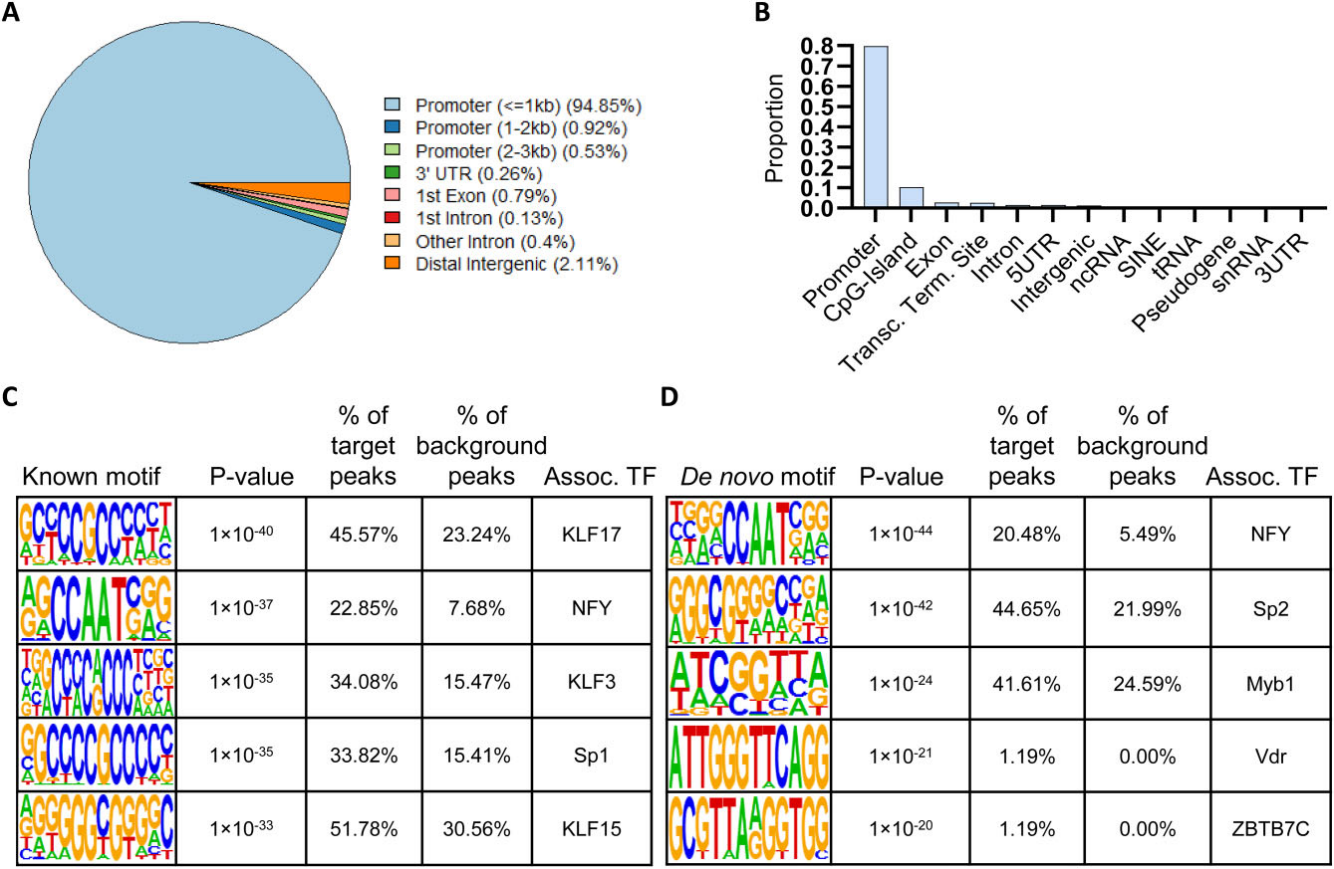
Consensus peaks of signal enrichment from untargeted CUT&Tag libraries derived from various cell lines colocalize with regulatory regions and G/C-rich sequence motifs. (**A**) Proportions of annotations overlapping consensus peaks (*n* = 2857) from complexity normalized, deduplicated untargeted CUT&Tag libraries (see Fig. 1B, bottom track) calculated by ChIPseeker. (**B**) Proportions of annotations overlapping consensus peaks (*n* = 2857) from deduplicated untargeted CUT&Tag libraries calculated by HOMER. (**C**) Top 5 known sequence motifs enriched in consensus peaks (*n* = 2857) from deduplicated untargeted CUT&Tag libraries calculated by HOMER alongside associated transcription factors (TF). *P*-values were calculated by binomial distribution test. (**D**) Top 5 *de novo* sequence motifs derived from consensus peaks (*n* = 2857) from deduplicated untargeted CUT&Tag libraries calculated by HOMER alongside associated TF. *P*-values were calculated by binomial distribution test.

**Figure 4.**
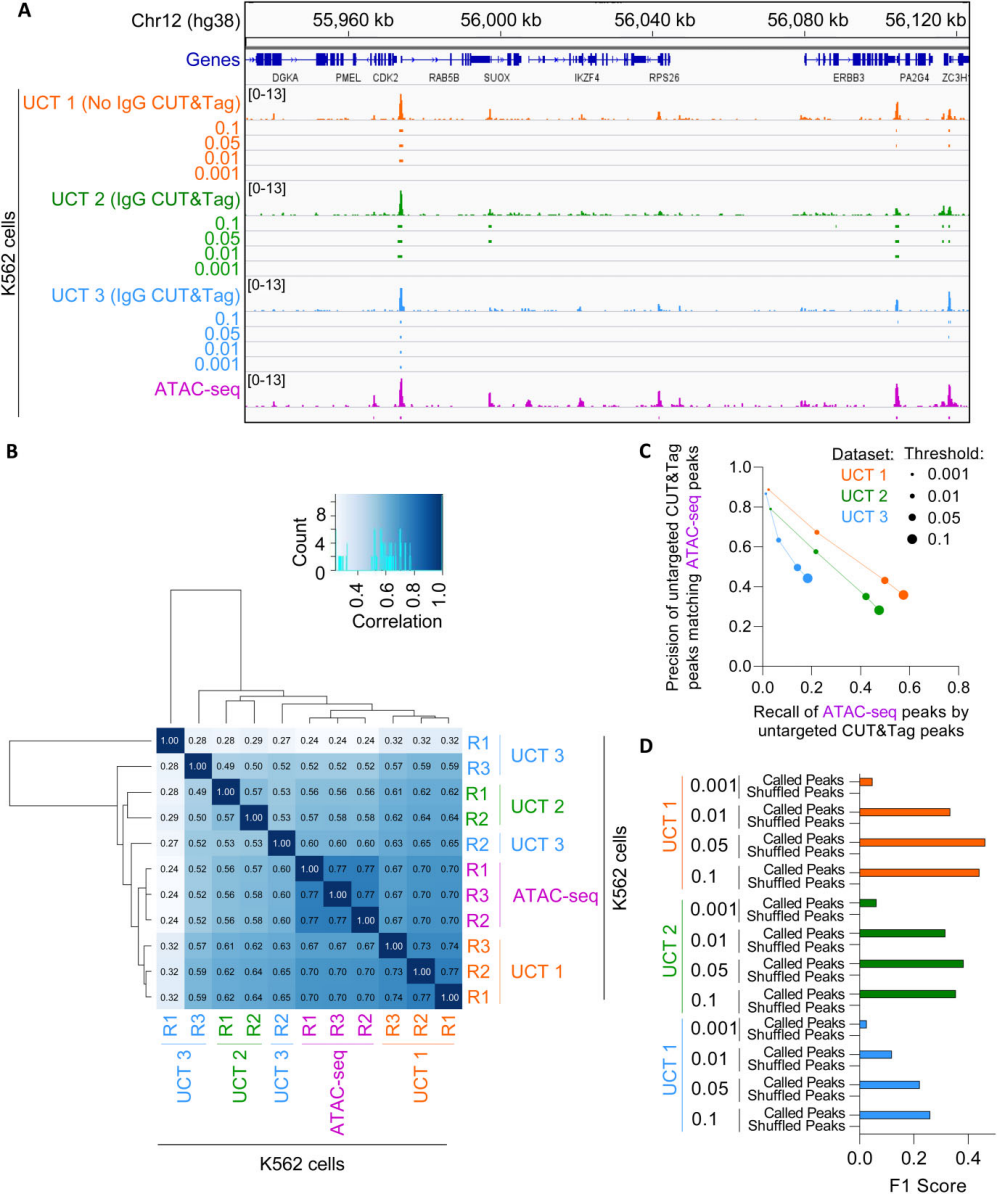
Untargeted CUT&Tag libraries correlate with genome-wide chromatin accessibility. (**A**) CPM-normalized read counts of complexity-normalized K562 cell-derived untargeted CUT&Tag experiment 1 (UCT 1; *n* = 3), untargeted CUT&Tag experiment 2 (UCT 2; *n* = 2), and untargeted CUT&Tag experiment 3 (UCT 3; *n* = 3) compared to complexity-normalized K562 cell-derived ATAC-seq libraries (*n* = 3), with untargeted CUT&Tag peaks called using SEACR without a negative control library at the indicated threshold at a representative locus on chromosome 12. (**B**) Clustered heatmap of Pearson’s correlation coefficients of normalized read counts from the complexity-normalized untargeted CUT&Tag libraries and ATAC-seq libraries across any site of signal enrichment of each sample. The correlation value distribution histogram is plotted within the Pearson’s correlation coefficient color scale (top center). (**C**) Precision/recall curves of SEACR-called peaks (using the indicated threshold) from the UCT 1, UCT 2, and UCT 3 untargeted CUT&Tag libraries in relation to the consensus peaks (*n* = 3) from the reference ATAC-seq libraries. (**D**) F1 scores of the SEACR-called consensus peaks (called using the indicated threshold) from the UCT 1 (*n* = 3), UCT 2 (*n* = 2), and UCT 3 (*n* = 3) untargeted CUT&Tag libraries or of shuffled peak controls as a measure of overlap with the consensus peaks (*n* = 3) from the reference ATAC-seq libraries.

We remained curious as to the characteristics of the observed genome-wide read distribution in the low-complexity untargeted CUT&Tag libraries, so we annotated the set of consensus peaks derived from the complexity-normalized and deduplicated libraries (Fig. 2B, bottom track). Given the use of Tn5 in the untargeted CUT&Tag reactions, we hypothesized that these peaks would be enriched with regard to regulatory elements, similar to what is observed for Tn5-catalyzed ATAC-seq data [52]. The consensus peaks from the complexity-normalized and deduplicated untargeted CUT&Tag libraries were strongly enriched within 1 kb of annotated promoters (Fig. 3A and B). Given that promoters have the propensity to form DNA secondary structures such as G4s [2], we wondered whether these untargeted CUT&Tag peaks colocalized with G4s. Identification of known sequence motifs that occur in untargeted CUT&Tag peaks revealed the prevalence of motifs with G- and C-rich sequences with tandem runs of guanines or cytosines (Fig. 3C). The untargeted CUT&Tag peaks contain sequences that match known binding motifs for the Sp1 transcription factor, a *bone fide* G4-binding protein [3, 4] and various related Krüppel-like transcription factors (KLF; Fig. 3C). Both known and *de novo* motifs possess TTA sequences and G-rich sequences (Fig. 3C and D), which have been previously identified at Tn5 insertion sites [80–82]. This suggests that the enrichment of reads in a low-complexity untargeted CUT&Tag library may be reflective of the accessibility of a given genomic locus. This observation of local accessibility being represented as off-target tagmentation events has been noted previously, but high salt is used during tagmentation to reduce off-target tagmentation at accessible chromatin [46, 47, 83]. However, under sub-optimal sample preparation conditions (i.e. cell clumping, low cell permeability, reduced salt concentrations, etc.) a background of untargeted tagmentation may be retained in the untargeted CUT&Tag libraries. To examine whether the genome-wide distribution of reads in untargeted CUT&Tag libraries correlates with chromatin accessibility, we compared the genome-wide enrichment of reads from three separate, multi-replicate untargeted CUT&Tag experiments performed in untreated K562 cells to an ENCODE project-derived ATAC-seq library (Supplementary Table S4) from untreated K562 cells. For clarity, we refer to the first untargeted CUT&Tag experiment as Untargeted CUT&Tag experiment 1 (UCT 1), the second as Untargeted CUT&Tag experiment 2 (UCT 2), and the third as Untargeted CUT&Tag experiment 3 (UCT 3). To ensure that the comparison of the untargeted CUT&Tag libraries and the more deeply sequenced ATAC-seq libraries was not strongly impacted by differences in sequencing depth and library complexity, the non-deduplicated libraries were downsampled to match the UCT 2 library with the limiting number of unique reads (Supplementary Table S5). Each library was then deduplicated, as recommended [79], prior to CPM normalization and peak calling. Given the sparse coverage of the CUT&Tag libraries (Fig. 4A), SEACR was utilized for peak calling for the CUT&Tag libraries, while MACS3 was utilized for the ATAC-seq libraries (Supplementary Table S5). Consensus peaks shared across all replicates were derived from these libraries, and normalized read counts from both untargeted CUT&Tag libraries were moderately correlated with the normalized ATAC-seq read counts when compared across all peaks of enrichment in any sample, with the exception of the first replicate of UCT 3 (Fig. 4B). To ensure that this correlation was not due to overly relaxed peak calling stringency, a precision-recall curve was constructed by comparing the overlap of triplicate-replicated ATAC-seq peaks to peaks called from the untargeted CUT&Tag libraries using the indicated thresholds from 0.001 to 0.1 (i.e. the top 0.1% of signal blocks to the top 10%) (Fig. 4C and Supplementary Table S6). Notably, the most stringently called peaks (0.001 threshold) from the untargeted CUT&Tag libraries overlapped the consensus ATAC-seq peaks with high precision. For example, a precision value of 0.9 would indicate that 90% of the untargeted CUT&Tag peaks from the indicated library overlapped any consensus ATAC-seq peak. High precision values thus indicate that the indicated proportion of peaks from the untargeted CUT&Tag experiment of interest match peaks from the reference ATAC-seq dataset. When called with a 0.001 threshold to retain the signal blocks with the top 0.1% of signal, 89% of the 97 consensus peaks from UCT 1, 79% of the 138 consensus peaks from UCT 2, and 87% of the 60 consensus peaks from UCT 3 overlapped one of the 3919 triplicate-replicated ATAC-seq peaks (Fig. 4C and Supplementary Table S6). Additionally, the less-stringently called peaks (0.1 threshold) from the untargeted CUT&Tag libraries matched the consensus ATAC-seq peaks with high recall. For example, a recall value of 0.8 would indicate that 80% of the consensus ATAC-seq peaks were overlapped by at least 1 peak from the indicated untargeted CUT&Tag library. High recall values thus indicate that the indicated proportion of the reference ATAC-seq dataset peaks are matched by at least one of the consensus peaks in the untargeted CUT&Tag experiment. Fifty-eight percent of the 3919 triplicate-replicated ATAC-seq peaks were overlapped by one of the 6426 consensus peaks from UCT 1, 48% by one of the 6692 consensus peaks from UCT 2, and 18% by one of the 1978 consensus peaks from UCT 3 (Fig. 4C and Supplementary Table S6), with UCT 3 peak recall being limited by the low number of consensus peaks in UCT 3 replicate 1 (Fig. 4B). These results indicate that peaks of enrichment of the untargeted CUT&Tag libraries are spatially correlated with peaks from the cell-line-matched ATAC-seq libraries, but the degree of precision or recall is dependent on peak calling parameters and peak numbers.

To ensure that these high precision and recall values were not inflated due to peak number imbalances between the peak sets, we compared similarity metrics between the ATAC-seq peaks and each set of called peaks, or between the ATAC-seq peaks and a control set of shuffled peaks, in which each peak was randomly redistributed throughout the genome to maintain the exact number of base pairs and peak widths present in each target peak set (Supplementary Table S6). When compared to the shuffled controls, the peaks called from the target libraries had much higher similarity measures to the ATAC-seq libraries based on the number of overlapping peaks (F1-score) (Fig. 4D) and number of base pairs overlapping (Jaccard index; Supplementary Table S6). Additionally, Fisher’s Exact Test deemed the association of the UCT 1 and UCT 2 library peaks with the ATAC-seq peaks to be significant, in contrast to the shuffled peaks with the same peak number imbalances (Supplementary Table S6). Consequently, we conclude that there is a nonrandom association between the genome-wide distributions of reads from the examined untargeted CUT&Tag libraries and ATAC-seq libraries, suggesting that the genome-wide signal enrichment for low-complexity CUT&Tag libraries is reflective of the local chromatin accessibility environment. This correlation between the untargeted CUT&Tag signal and chromatin accessibility also likely explains some of the variability observed in the genome-wide distribution of reads and signal amplitudes from the untargeted CUT&Tag libraries examined in Figs 1–3 as they were generated from a variety of human cell lines (Supplementary Table S1), which would have cell-type-specific differences in chromatin accessibility [84].

### Low-complexity untargeted CUT&Tag libraries colocalize with G4s

**Figure 5.**
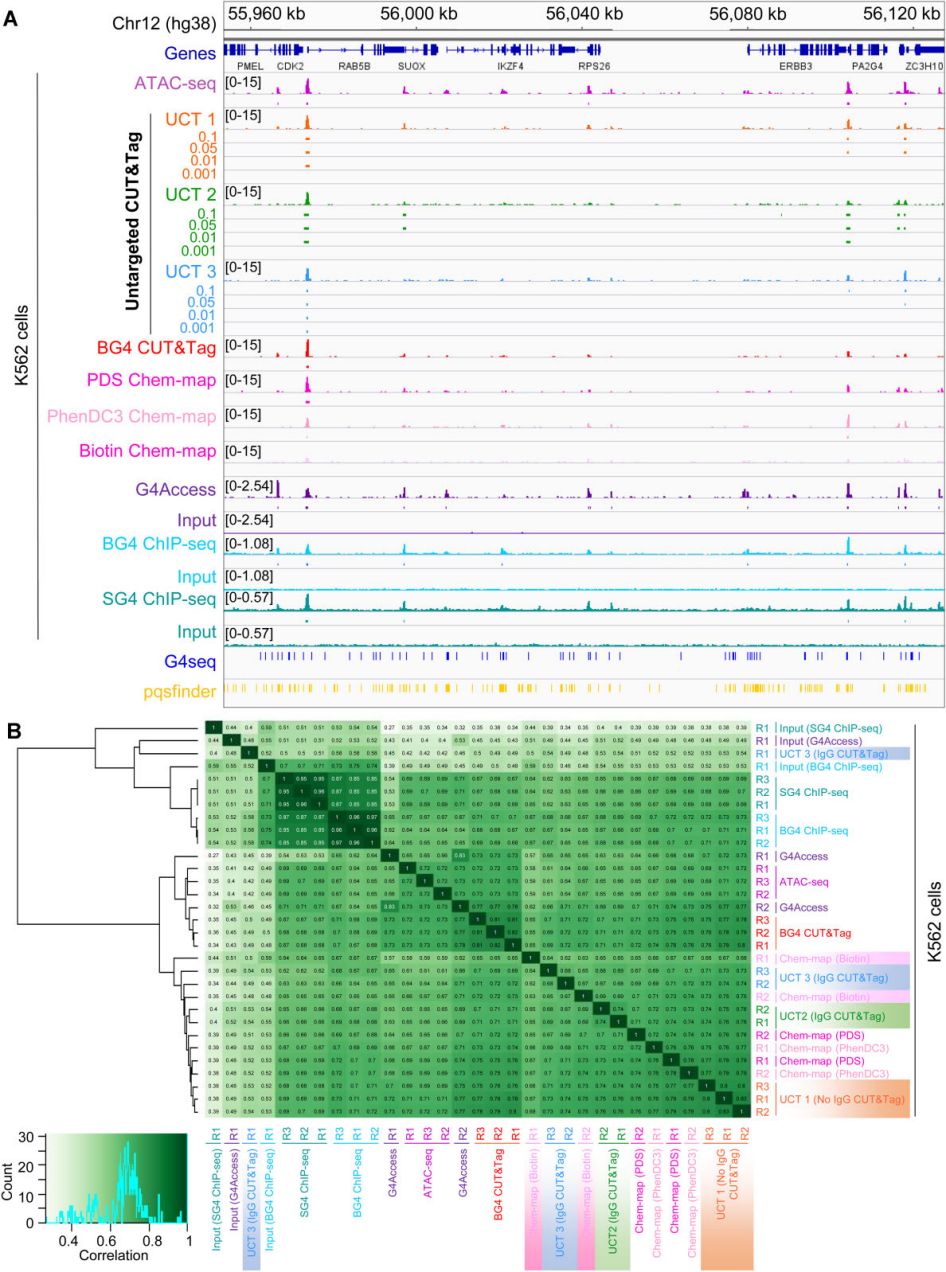
Untargeted CUT&Tag libraries correlate with genome-wide maps of G-quadruplex (G4) presence and chromatin accessibility. (**A**) CPM-normalized read counts of K562 cell-derived libraries and called peaks at representative locus on chromosome 12. Libraries of untargeted CUT&Tag 1 (UCT 1; *n* = 3), untargeted CUT&Tag library 2 (UCT 2; *n* = 2), untargeted CUT&Tag library 3 (UCT 3; *n* = 3), ATAC-seq (*n* = 3), BG4 CUT&Tag libraries and matched IgG CUT&Tag controls (*n* = 3), PDS- or PhenDC3-targeted Chem-map libraries and matched biotin-targeted ChemMap controls (*n* = 2), G4Access and matched input controls (*n* = 2), BG4 ChIP-seq and matched input controls (*n* = 3), SG4 ChIP-seq and matched input controls (*n* = 3), G4-seq *in vitro* mapped G4-forming sequences, and pqsfinder-predicted G4-forming sequences were plotted on the indicated axes, with samples matched to respective controls. Tn5-derived libraries (ATAC-seq, CUT&Tag, and Chem-map) were normalized for library complexity by downsampling, and all libraries were deduplicated prior to read count averaging across replicates and peak calling. (**B**) Clustered heatmap of Pearson’s correlation coefficients of normalized read counts from the above libraries across any site of signal enrichment of each sample. The correlation value distribution histogram is plotted within the Pearson’s correlation coefficient color scale (bottom left). Tn5-derived untargeted CUT&Tag libraries are indicated by shaded rectangles.

Following from our observation of the association of untargeted CUT&Tag peaks with promoters, Tn5-accessible chromatin, and G-rich sequences, we next investigated the genome-wide association between untargeted CUT&Tag reads and G4-forming loci. The UCT 1, UCT 2, and UCT 3 untargeted CUT&Tag libraries were compared to BG4 CUT&Tag libraries [44], BG4 ChIP-seq libraries [85], G4Access libraries [41], PDS and PhenDC3 Chem-map libraries [45], and SG4 ChIP-seq libraries [36], all derived from K562 cells, in addition to predicted G4s (pqsfinder) [86] and to G4s that fold *in vitro* in single-stranded DNA (G4-seq) [34] (Supplementary Table S7). To ensure that the library complexity was normalized between the Tn5-derived untargeted CUT&Tag libraries and the other Tn5-derived libraries, (i.e. BG4 CUT&Tag libraries and Chem-map libraries), these libraries were downsampled to match the library with the limiting number of unique reads, while leaving reads from the non-Tn5-derived libraries unmodified except for deduplication (Supplementary Table S8). Following peak calling for each library, the normalized read count correlations were compared at all sites of enrichment for each sample across the genome (Fig. 5A). Notably, input samples from sonicated genomic DNA (for ChIP-seq and G4Access) were the least correlated with the Tn5 libraries (Fig. 5B) due to the relatively low signal-to-noise ratio (Supplementary Table S8) and low local enrichment, appearing as flat, unenriched signals when plotted against their matched samples (Fig. 5A). The BG4 and SG4 ChIP-seq libraries also strongly correlated due to high similarity and similar signal-to-noise ratios (Fig. 5B and Supplementary Table S8). The G4Access libraries correlated most strongly with the ATAC-seq library, serving as a good validation of matching accessible chromatin signals across the cell-line-matched libraries (Fig. 5B). The G4Access libraries also correlated with the BG4- and SG4-targeted ChIP-seq libraries and with the Tn5-based G4 mapping methods of BG4 CUT&Tag and Chem-map (Fig. 5B).

Additionally, analysis of other publicly available G4- and i-motif-mapping libraries from HEK293T cells enabled the validation of other orthogonal G4 mapping methods; HEK293T libraries generated by G4P ChIP-seq [37], SG4 CUT&Tag [5], BG4 CUT&Tag [5, 30, 43], PDS Chem-map [5], and i-motif-mapping iMab CUT&Tag [30] were processed as earlier (Supplementary Tables S9 and S10) and compared with similar high correlations (Supplementary Fig. S4). Notably, the G4 and i-motif mapping results were again strongly correlated with the respective cell line-matched ATAC-seq libraries and Omni-ATAC-seq library (i.e. an improved cell-line-agnostic ATAC-seq protocol [87]), indicating that G4s and i-motifs appear to be present in close to all, if not all, open chromatin regions examined. The consistent correlations between the Tn5-based methods of G4 and i-motif mapping (BG4 CUT&Tag, SG4 CUT&Tag, iMab CUT&Tag, Chem-map), ChIP-seq-based methods of G4 mapping (BG4 ChIP-seq, SG4 ChIP-seq, G4P ChIP-seq), and other orthogonal G4 mapping methods (G4Access), coupled with the biological validation performed in each assay prior to sequencing, suggest that the reproducible genome-wide signal enrichment observed across these varied DNA secondary structure mapping methods (Fig. 5 and Supplementary Fig. S4) is reflective of the genome-wide distribution of G4s and i-motifs.

However, we did observe that low-complexity libraries from the untargeted control libraries for the Tn5-based methods were similarly correlated and interspersed among the targeted G4 and i-motif mapping libraries following clustering (Fig. 5B; shaded rectangles). Among the K562 cell-derived libraries, these samples included non-targeting IgG CUT&Tag (i.e. UCT 2, shaded in green, and UCT 3, shaded in blue), no IgG CUT&Tag (i.e. UCT 1, shaded in orange), and Chem-map with biotin as the target (shaded in pink). Similarly, the SG4 R105A CUT&Tag controls for SG4 CUT&Tag (using the R105A variant of SG4 with minimized ability to bind G4s [36]) from the HEK293T libraries were highly correlated with the other Tn5-derived samples (Supplementary Fig. S4B). However, there is a slight decrease in FRiP score for the untargeted CUT&Tag libraries when compared to the G4-targeted CUT&Tag libraries (Supplementary Tables S8 and S10), indicating that targeting of Tn5 to G4s does seem to have at least somewhat increased specificity for mapping G4s. Additionally, it is notable that the non-targeting IgG CUT&Tag library from HEK293T cells (SRR14879749; Supplementary Fig. S4) shared very little correlation with the other Tn5-derived libraries, showing that certain technical factors may allow for creation of high-complexity, poorly enriched untargeted CUT&Tag controls that appear flat when compared to matched G4-targeted CUT&Tag libraries (Supplementary Fig. S4A). Of note, the matched untreated BG4 CUT&Tag libraries and Omni-ATAC-seq libraries (but not the DMSO- and PDS-treated BG4 CUT&Tag libraries) from this publication [43] also have reduced correlation with other samples from untreated HEK293T cells (Supplementary Fig. S4B), suggesting that other technical variations may have occurred that influenced the genome-wide signal enrichment observed in these libraries. Overall, we observe a reproducible genome-wide signal enrichment across libraries prepared from Tn5-based G4-mapping techniques, G4Access, ATAC-seq, and low-complexity Tn5-based negative controls (IgG CUT&Tag, untargeted CUT&Tag, biotin Chem-map, and SG4 R105A CUT&Tag) (Fig. 5 and Supplementary Fig. S4), suggesting that background Tn5 activity at accessible chromatin regions results in a reproducible signal at these regions in low-complexity, but not high-complexity, negative control Tn5-derived libraries.

**Figure 6.**
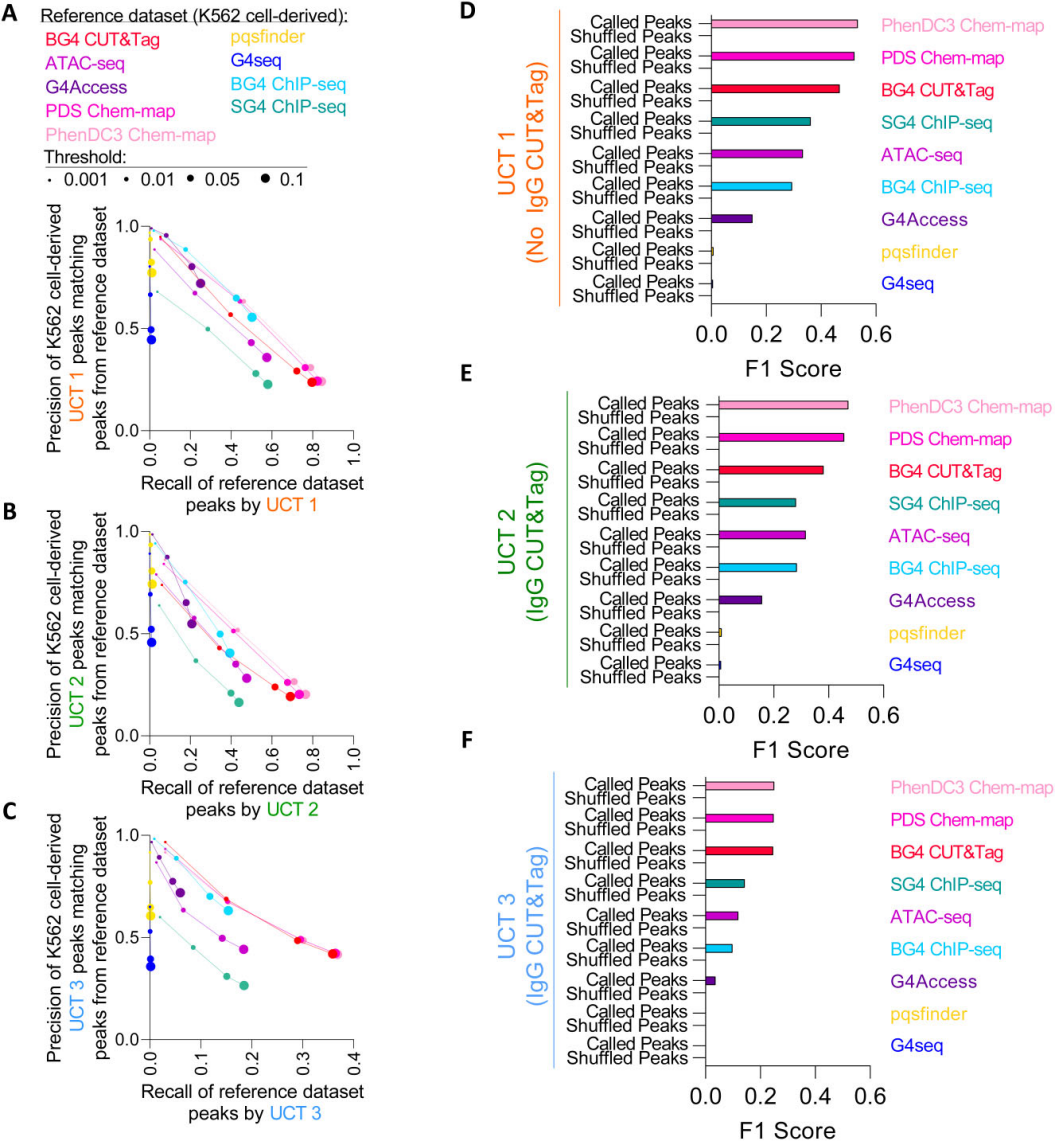
Peaks called from untargeted CUT&Tag libraries overlap peaks from G4-mapping techniques and ATAC-seq with high precision and recall, depending on the SEACR peak calling threshold. (**A**) Precision recall plot of consensus peaks from the UCT 1 (No IgG CUT&Tag) experiment from K562 cells with reference to the indicated reference G4-mapping library (by color), with SEACR peak calling thresholds for UCT 1 indicated by dot size. (**B**) Precision recall plot of consensus peaks from the UCT 2 (non-targeting IgG CUT&Tag) experiment from K562 cells as described in panel (A). (**C**) Precision recall plot of consensus peaks from the UCT 3 (non-targeting IgG CUT&Tag) experiment from K562 cells as described in panel (A). (**D**) F1 scores of consensus peaks (SEACR, 0.01 threshold) or shuffled consensus peaks from UCT 1 with respect to the indicated G4-mapping library. (**E**) F1 scores of consensus peaks from UCT 2 with respect to the indicated G4-mapping library, as described in panel (D). (**F**) F1 scores of consensus peaks from UCT 3 with respect to the indicated G4-mapping library, as described in panel (D).

To quantify the observed associations between the low-complexity untargeted CUT&Tag libraries and the Tn5-derived G4-mapping libraries, we again constructed precision/recall curves by comparing peaks called in the untargeted CUT&Tag libraries to peaks called from G4-mapping libraries (Fig. 6A–C and Supplementary Table S11). Stringently called peaks (0.001 threshold) from the UCT 1, UCT 2, and UCT 3 libraries had high precision at matching peaks in the G4-mapping libraries, and recall values from less stringently called peaks (0.1 threshold) in the UCT 1, UCT 2, and UCT 3 libraries were especially high for the peaks from the complexity-matched Tn5-derived G4 mapping libraries (i.e. BG4 CUT&Tag, PDS Chem-map, and PhenDC3 Chem-map) with a similar number of called peaks. Additionally, we measured high precision and recall of the untargeted CUT&Tag libraries with peaks called from ATAC-seq libraries and peaks called from BG4 ChIP-seq libraries. The high number of peaks called in the G4Access libraries limited overall recall of the G4Access peaks, but the untargeted CUT&Tag libraries had high precision in matching the peaks called from G4Access. Conversely, the relatively noisy SG4 ChIP-seq library had a lower number of called peaks, limiting the overall precision of the peaks from UCT 1, UCT 2, and UCT 3, but boosting the recall values. The genomic intervals of G4s from the *in vitro* G4-seq library and the algorithmically predicted pqsfinder library were matched with high precision by the untargeted CUT&Tag libraries, but recall was limited, given the large imbalance between the numbers of genomic intervals present in these libraries. This recall limitation is also reflective of the fact that not all algorithmically predicted and *in vitro-*observed G4s will form *in cellulo*. Thus, low-complexity untargeted CUT&Tag libraries have high precision and recall of peaks from G4-mapping and ATAC-seq libraries, indicating a nonrandom genome-wide pattern of colocalization.

To ensure that these precision and recall values were not reflective of random overlaps between large numbers of peaks throughout the genome, we examined the peak-overlap-derived F1 scores, base-pair-overlap-derived Jaccard indices, and derived statistical significance using Fisher’s exact test using peaks called by SEACR with a 0.01 threshold (Supplementary Table S11). The F1 scores (Fig. 6D–F) and Jaccard indices of the UCT 1, UCT 2, and UCT 3 libraries were much higher than those of shuffled control peaks and were statistically significant when measured with a Fisher’s exact test (Supplementary Table S11). Collectively, we interpret these results as evidence of a nonrandom association of untargeted CUT&Tag signal enrichment from low-complexity libraries with signal enrichment from libraries produced from G4-mapping methods and ATAC-seq, although we note that counterexample libraries (e.g. SRR14879749) do exist. However, the high degree of correlation between the low-complexity untargeted CUT&Tag libraries and the cell-line-matched G4- and i-motif-targeted CUT&Tag libraries led us to wonder whether G4 identification in G4-targeted CUT&Tag libraries would be impacted by the inclusion of a matched low-complexity untargeted CUT&Tag library.

### Detection of G4s using differential enrichment analysis between matched G4-targeted and untargeted CUT&Tag libraries impairs recall when mapping G4s

**Figure 7.**
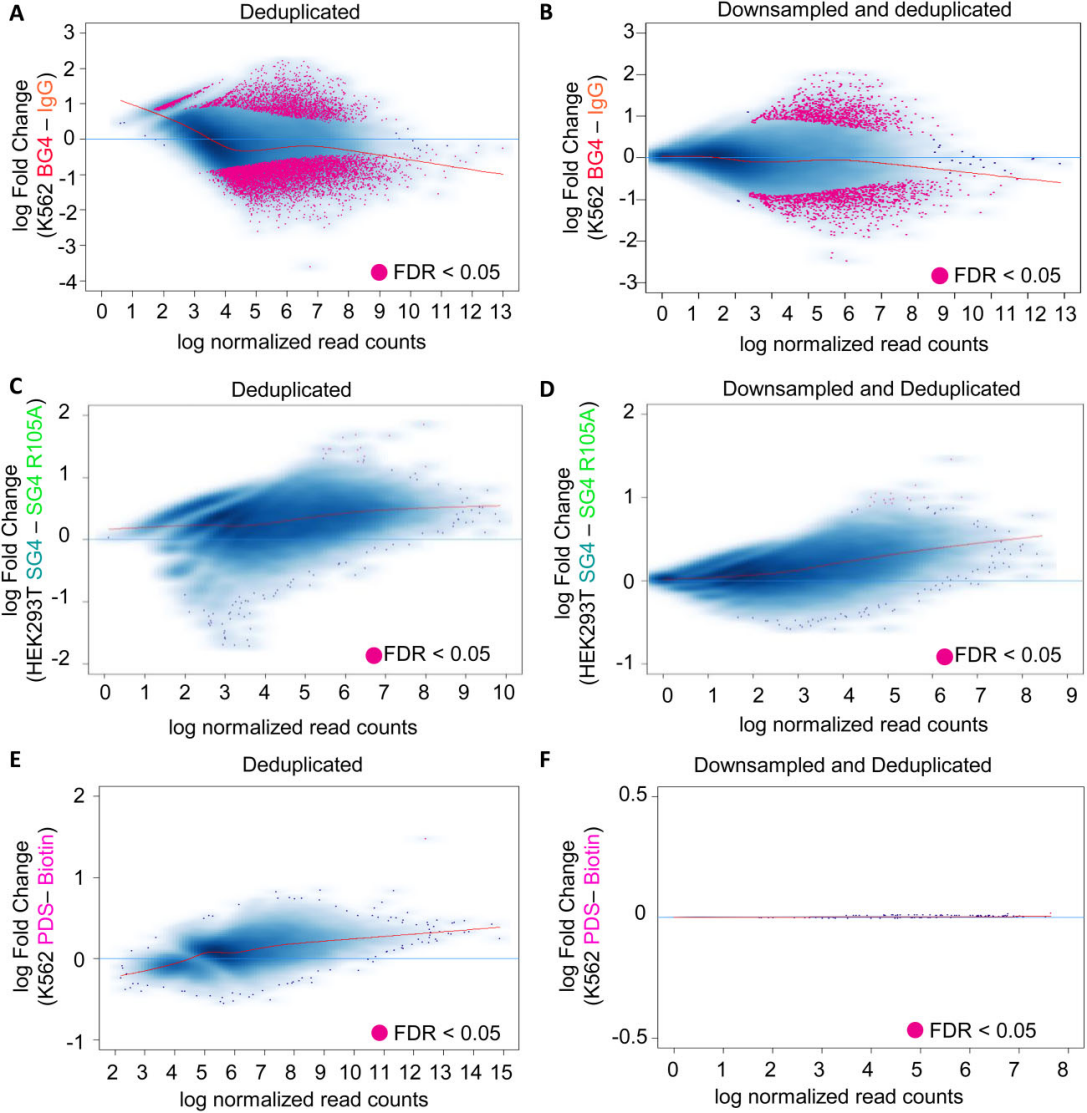
Normalized read counts of G4-mapping CUT&Tag libraries at SEACR-called peaks of signal enrichment have variable differential enrichment compared to matched untargeted CUT&Tag libraries. (**A**) Log fold changes of DEseq2-normalized read counts of deduplicated K562 cell-derived BG4 CUT&Tag libraries (from [44]) (*n* = 3) and matched IgG controls (*n* = 3) at any site of enrichment called in each sample with a 0.01 SEACR threshold. Points with a significant (FDR < 0.05) fold change between the samples are labeled in magenta, with a locally estimated scatterplot smoothing (LOESS) fit plotted in red. 8679 sites had significant fold changes in read counts. (**B**) Log fold change of normalized read counts as in panel (A), but with libraries downsampled to normalize library complexity prior to DEseq2 normalization. 2235 sites had significant fold changes in read counts. (**C**) Log fold changes of DEseq2-normalized read counts of deduplicated HEK293T cell-derived SG4 CUT&Tag libraries (from [5]) (*n* = 3) and matched SG4 R105A CUT&Tag controls (*n* = 2) at any site of enrichment called in each sample with a 0.01 SEACR threshold. Points with a significant (FDR < 0.05) fold change between the samples are labeled in magenta, with a LOESS fit plotted in red. Fifteen sites had significant fold changes in read counts. (**D**) Log fold change of normalized read counts as in panel (C), but with libraries downsampled to normalize library complexity prior to DEseq2 normalization. Sixteen sites had significant fold changes in read counts. (**E**) Log fold changes of DEseq2-normalized read counts of deduplicated K562 cell-derived PDS Chem-map libraries (from [45]) (*n* = 2) and matched biotin Chem-map libraries (*n* = 2) at any site of enrichment called in each sample with a 0.01 SEACR threshold. Points with a significant (FDR < 0.05) fold change between the samples are labelled in magenta, with a LOESS fit plotted in red. One site had significant fold change in read counts. (**F**) Log fold change of normalized read counts as in panel (E), but with libraries downsampled to normalize library complexity prior to DEseq2 normalization. One site had a significant fold change in read counts.

We hypothesized that G4s in G4-mapping CUT&Tag libraries could be identified by determining peaks with a significant (FDR < 0.05) fold change of signal in the G4-targeted CUT&Tag libraries when compared to the appropriate matched untargeted CUT&Tag control libraries. To examine this, we utilized DiffBind [70, 71] to perform DEseq2-mediated [72] relative log expression normalization on either the deduplicated libraries or complexity-normalized (i.e. downsampled then deduplicated) libraries of interest. When comparing peaks of enrichment across both matched G4-targeted and untargeted CUT&Tag libraries from K562 cells [44], 8679 sites were differentially enriched between the two sets of libraries, with 2543 peaks having a significant positive fold change of normalized read count signal in the G4-targeted CUT&Tag libraries when compared to the untargeted CUT&Tag libraries (Fig. 7A and Supplementary Table S12). However, when the libraries were normalized to account for library complexity differences prior to further DESeq2-mediated relative-log expression normalization, the number of sites with a significant positive fold change in the G4-targeted CUT&Tag libraries when compared to the untargeted CUT&Tag libraries was reduced to only 1008 sites (Fig. 7B and Supplementary Table S12). When this method of differential analysis was completed for the matched G4-targeted SG4 CUT&Tag libraries with respect to the control SG4 R105A CUT&Tag libraries from HEK293T cells [5], even fewer differentially enriched peaks were identified in deduplicated (Fig. 7C; 15 differentially enriched peaks) or complexity-normalized libraries (Fig. 7D; 16 differentially enriched peaks). A similar, but more striking result was observed when applying this analysis to the biotinylated PDS Chem-map libraries and the matched biotin control Chem-map libraries from K562 cells [45], where only one site was observed to have differential enrichment in the biotinylated PDS-targeted Chem-map libraries when compared to the biotin control Chem-map libraries (Fig. 7E and F). These results indicate that use of differential read count enrichment analysis to identify G4s in Tn5-derived G4-mapping libraries will impair identification of potential G4s in certain libraries (i.e. reducing false positives while simultaneously increasing false negatives) due to substantial overlap of read enrichment at the same loci in both G4-targeted and low-complexity untargeted CUT&Tag libraries.

### Use of matched untargeted control libraries when calling peaks of signal enrichment in Tn5-derived G4-mapping libraries improves precision but limits recall when identifying potential G4s

**Figure 8.**
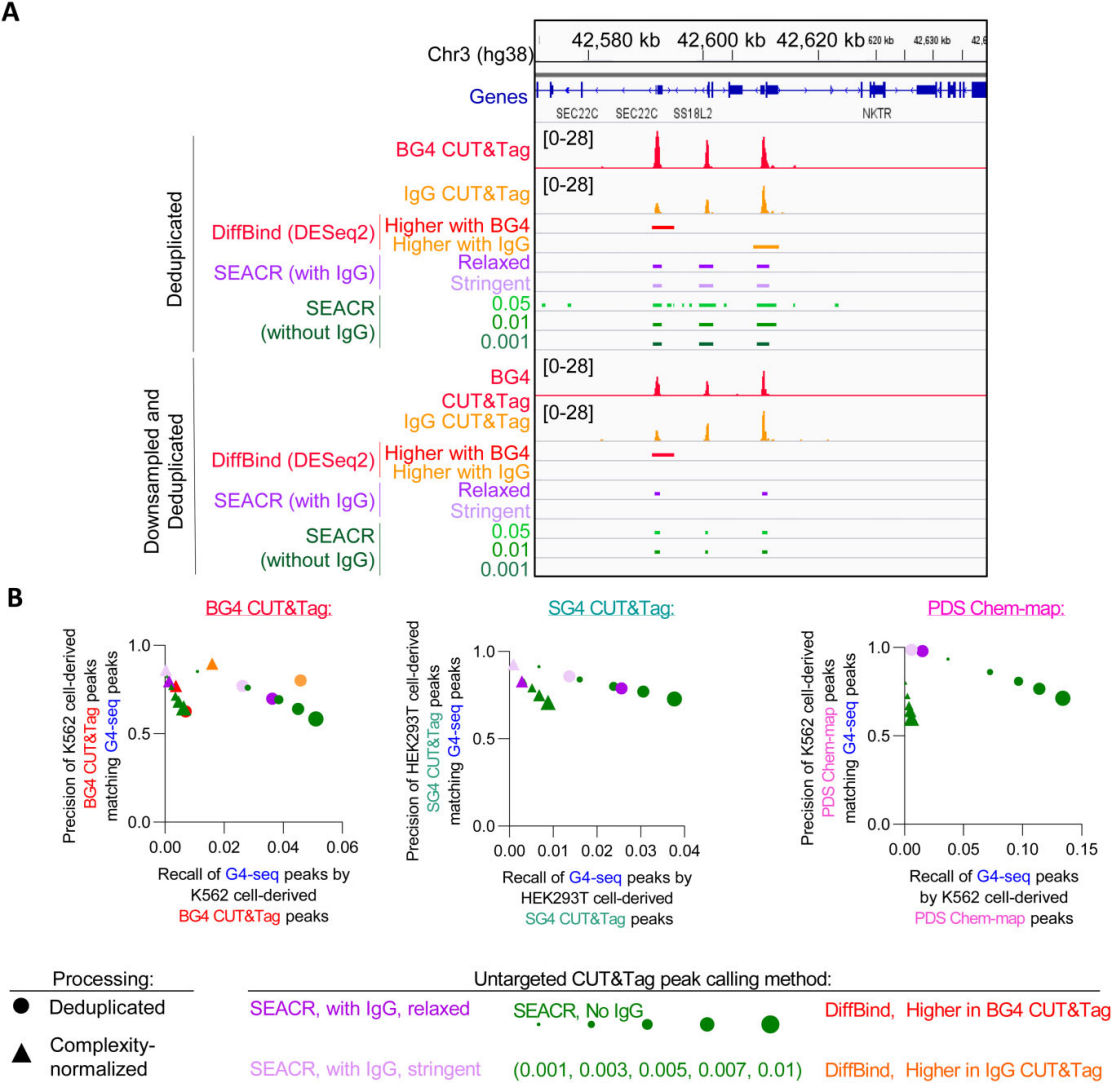
Use of matched untargeted CUT&Tag libraries when calling peaks in Tn5-derived G4-mapping libraries limits recall of potential G4s. (**A**) Average K562 cell-derived BG4 CUT&Tag (*n* = 3) and IgG CUT&Tag (*n* = 3) normalized read counts at a representative locus on chromosome 3, illustrating peaks called using different methods. (**B**) Precision-recall plots of replicate-conserved peaks called from K562 cell-derived BG4 CUT&Tag (*n* = 3), HEK293T cell-derived SG4 CUT&Tag (*n* = 3), and K562 cell-derived PDS Chem-map (*n* = 2) libraries when compared to a reference G4-seq library.

This overlap of read enrichment in G4-targeted and untargeted CUT&Tag libraries and ATAC-seq libraries suggests that the peaks called by the Tn5-based G4 mapping methods may simply be maps of accessible chromatin instead of G4s. However, prior biological and biochemical validation of each G4 mapping technique (e.g. siRNA knockdown of G4 resolving helicases of G4Access [41], CRISPR-mediated mutation of the endogenous MYC G4 when validating SG4 CUT&Tag [5], establishment of specificity of probes for G4 binding *in vitro* [35, 36]) when combined with the correlations noted earlier between the Tn5-based and non-Tn5-based G4-mapping methods suggests that these techniques do identify *bona fide* G4-forming loci. Interestingly, the matched low-complexity negative controls for the Tn5-based methods generated and illustrated in the prior studies [5, 44, 45] were not utilized when peak calling for Chem-map [5, 45], SG4 CUT&Tag [5], or BG4 CUT&Tag [44], although the high-complexity SRR14879749 library was used against its matched BG4 CUT&Tag library for peak calling [43]. We were curious as to what effect use of the low-complexity untargeted CUT&Tag libraries would have when calling peaks in matched G4-targeted CUT&Tag libraries using the SEACR [49] algorithm, in contrast to the SEACR threshold-based method of G4 peak identification in the literature [5, 44, 45]. We reasoned that the signal enrichment overlap between the untargeted CUT&Tag libraries and the G4-targeted CUT&Tag libraries could limit recall of potential G4s while improving precision by eliminating false-positive peaks at Tn5-accessible loci that lack a folded G4. To test this, we compared the precision and recall of peaks called with or without the appropriate matched untargeted CUT&Tag libraries from BG4 CUT&Tag [44], PDS Chem-map [45], and SG4 CUT&Tag [5] libraries to peaks called from G4-seq [34], representing loci that can form G4s *in vitro*. Representative peaks identified through these methods or through differential enrichment analysis (Fig. 7A and B) for the BG4 CUT&Tag library were visualized at a representative locus (Fig. 8A) to illustrate the differences in identified peaks using each peak calling method with or without complexity normalization (i.e. downsampling and deduplication) between the targeted and untargeted libraries. When precision and recall of the G4-mapping libraries for mapping the G4-seq-observed G4s [34] were quantified (Supplementary Table S12), we observed that the choice of SEACR threshold utilized when peak calling without an untargeted CUT&Tag dataset determined a trade-off between precision and recall when mapping G4s (Fig. 8B and Supplementary Fig. S5). Of course, recall of the G4-seq-observed sequences that can form G4s *in vitro* will be limited by cell-type- and loci-specific factors such as chromatin openness, supercoiling, and protein binding in a cellular environment, and precision/recall statistics are also impacted by peak number imbalances. Reflecting this, peaks of BG4 CUT&Tag called by using the most relaxed SEACR threshold tested (0.01; 19 940 peaks, Supplementary Table S12) retained decent (>0.5) precision when matching G4-seq G4s (427 833 peaks, Fig. 8B, Supplementary Table S12), but precision of these same peaks (*n* = 19 940) for matching cell-line-matched BG4 ChIP-seq-mapped G4s (7777 peaks, Supplementary Table S12) was lower, likely due to the peak number imbalance between these peak sets. However, use of the matched untargeted CUT&Tag libraries in the SEACR algorithm (instead of specifying a numeric threshold) in both stringent and relaxed stringency modes had the effect of reducing recall of potential G4s with respect to peaks called when using the most relaxed SEACR threshold tested (0.01; Fig. 8B, Supplementary Table S12) while slightly improving precision of peaks overlapping a G4-seq observed G4. When the same analysis was applied comparing cell-line-matched BG4 CUT&Tag and PDS Chem-map libraries to BG4 ChIP-seq data, a similar result was observed, with use of the untargeted CUT&Tag library when peak calling leading to a reduction in recall and improvement in precision (Supplementary Fig. S5). These impacts of limiting recall and improving precision were consistent across the libraries examined, although the precise effects on precision and recall had varying magnitudes based on the specific libraries examined (Fig. 8B and Supplementary Fig. S5). For example, peak calling of the PDS Chem-map dataset using the matched untargeted biotin Chem-map samples severely limited recall, and no consensus (*n* = 2) peaks were called following downsampling (Fig. 8B and Supplementary Table S12). Complexity normalization of the targeted libraries to the untargeted libraries prior to peak calling did not change these outcomes (Fig. 8B and Supplementary Fig. S5), indicating that this limitation in recall remains after normalizing complexity differences between the targeted and untargeted CUT&Tag libraries. These results indicate that use of untargeted CUT&Tag libraries for peak calling can reduce the number of previously mapped G4s identified (i.e. lower recall) while increasing the precision of only calling previously mapped G4s as peaks when compared to mapping G4s using only the SEACR threshold parameter. This analysis also demonstrates that the use of SEACR without an untargeted CUT&Tag library can be used for calling peaks from Tn5-derived G4-mapping libraries, but the threshold chosen impacts precision and recall of G4 mapping. These results suggest that stringent protocol optimization should be undertaken to produce high-complexity untargeted CUT&Tag libraries and reduce any untargeted CUT&Tag tagmentation and accessibility-driven read enrichment prior to sequencing, or orthogonal methods and/or biological validation could be utilized to ensure minimal interference of signal from untargeted tagmentation when mapping G4s with Tn5-based methods.

## Discussion

The need to predict and map G4s (reviewed in [29]) has spurred technical innovation, and a diverse suite of G4 mapping tools exists to meet multiple experimental needs. For example, full-length antibodies [88], single-chain antibodies [35], nanobodies [36], protein-based probes [37], and G4-ligand-based probes [45] have been utilized for chromatin immunoprecipitation [2, 89] and *in situ* Tn5-based sequence mapping methods [44, 45, 90], alongside the MNase-catalyzed G4Access method [41]. While each method has a unique set of nuances and limitations, CUT&Tag has been noted for its high signal-to-noise ratio and low input requirements [46], which can enable G4 mapping of single-cell populations [44] and precious patient-derived samples. However, it has been previously noted that CUT&Tag analyses can be influenced by untargeted Tn5 cutting at accessible chromatin [46, 47, 50, 51]. Additionally, simply by reducing the ionic strength of the buffer during tagmentation, Tn5-based cleavage can be used to map accessible chromatin in the vicinity of antibody targets, rather than solely mapping the site of antibody binding [83]. Given the dynamic nature of G4s and their high degree of overlap with accessible regulatory chromatin, it is important to characterize which G4 mapping methods and bioinformatics analyses can be used to distinguish the presence or absence of G4s without mapping accessible chromatin environments instead.

Our initial correlation analysis between the untargeted CUT&Tag libraries unsurprisingly revealed a pattern of enrichment at Tn5-accessible regulatory chromatin such as promoter regions. However, the only modest correlations that we observed between the genome-wide signal distributions across all of the libraries examined can potentially be explained by differences in chromatin accessibility between the various cell lines from which the libraries were generated. To control for this variability, we compared cell-line-matched G4- and i-motif-mapping libraries to untargeted CUT&Tag libraries. These comparisons revealed high correlations between Tn5-accessible chromatin mapped by ATAC-seq, genome-wide signal enrichment in untargeted CUT&Tag libraries, and chromatin containing G4- and i-motif secondary structures mapped by a variety of methods. The high degree of overlap between accessible chromatin and G-quadruplex DNA structures has been consistently reported in prior studies [2, 4, 30, 41, 85]. Based on the strong observed correlation of G4-targeted CUT&Tag assays and ATAC-seq, this suggests that the vast majority of accessible chromatin regions may contain mappable G4s and/or i-motifs. However, the association between G4s and accessible chromatin has been complicated by difficulty establishing causality of G4s in promoting and/or maintaining open chromatin. Recent studies have presented observations that suggest that the presence of a folded G4 at a locus can prevent nucleosome deposition. For example, mutation of the endogenous *MYC* promoter G4 to prevent G4 formation leads to *de novo* nucleosome deposition at the locus [5], and insertion of a G4-containing promoter from the mouse genome into human cell lines resulted in nucleosome exclusion at the locus, whereas the matched G4-mutated promoter construct had higher nucleosome occupancy at the same locus [91].

This strong connection between G4s and accessible chromatin suggests that the untargeted CUT&Tag signal enrichment that we and others observe at G4-forming loci could be reflective of three possibilities. First, the accessible chromatin environment due to the nucleosome exclusion capabilities of G4s would promote local Tn5 tagmentation. Second, other literature has implicated both hyperactive Tn5 [92] and Protein A [93] in binding directly to G4s, and this direct G4-Protein A-Tn5 interaction would promote local Tn5 tagmentation, rather than the local accessibility environment. Third, local Tn5 tagmentation could be promoted by chromatin accessibility in the absence of a folded G4. An intriguing test of these different models could be performed at an engineered genomic locus lacking or containing a G4-forming sequence that retains accessibility in the absence of a folded G4; if untargeted CUT&Tag and ATAC-seq libraries both have signal enrichment at this open chromatin locus when the G4-forming sequence is mutated to prevent G4 formation while targeted G4 mapping methods do not, this could clarify the origin of the untargeted CUT&Tag signal. A similar experiment has been performed, but without the inclusion of ATAC-seq libraries. When the endogenous *MYC* promoter G4-forming sequence is mutated to abrogate G4 formation, BG4 CUT&Tag signal, PDS Chem-map signal, and SG4 CUT&Tag signals are still observed at the *MYC* promoter, although the signal intensity is visibly reduced compared to the same assays performed in cells containing the wild-type *MYC* promoter G4 or the *KRAS* promoter G4 [5]. Additionally, a slight enrichment of G4-interaction-deficient SG4 R105A CUT&Tag signal is also present at this site [5], but it is difficult to know if this signal would not match that of wild-type SG4 CUT&Tag if the SG4 R105A CUT&Tag libraries were sequenced to the same depth as the wild-type SG4 CUT&Tag libraries. Given our observation of higher FRiP scores of the G4-targeted CUT&Tag libraries when compared to the matched untargeted CUT&Tag libraries and given the difference in DNA yield when using a G4-targeted probe for CUT&Tag, we conclude that a certain proportion of signal enrichment at a given locus in a G4-targeted CUT&Tag reaction is due to G4-targeted tagmentation. However, the observation of remaining CUT&Tag signal enrichment at the mutated *MYC* promoter G4 locus in the targeted CUT&Tag libraries [5] suggests that at least some proportion of the signal enrichment in G4-targeted CUT&Tag at a G4 could originate from tagmentation of accessible chromatin in addition to targeted tagmentation at the G4, with similar effects expected when mapping i-motifs using iMab CUT&Tag. This leads to the conclusion that G4-targeted CUT&Tag signal enrichment is reflective of an additive combination of both G4-targeted and untargeted tagmentation. However, the relative contributions of G4s and accessible chromatin to G4-targeted CUT&Tag signal amplitude at a given locus have not been defined except at the *MYC* promoter [5], which points toward the need for further biological validation of CUT&Tag-mapped G4s.

Use of differential enrichment analyses to identify G4s in G4-targeted CUT&Tag libraries is complicated by the simultaneous enrichment of both the G4-targeted tagmentation and the untargeted tagmentation at accessible chromatin. Various normalization methods exist to take into account differences in sequencing depth, duplication rates, and changes in signal variation based on signal intensity. When comparing matched targeted and untargeted datasets created with a uniform source of Tn5, normalization of library sizes is suggested based on carryover of *E. coli* DNA remaining following Tn5 expression and purification [78]. However, certain commercial suppliers of Tn5 recommend against utilization of this method, claiming that the residual *E. coli* DNA content is miniscule following purification of Tn5. To avoid any bias from differing *E. coli* DNA content when comparing cell-line matched libraries, we trialed both deduplication and complexity normalization (downsampling and deduplication [79]) prior to DEseq2-mediated normalization for differential enrichment analysis. Notably, complexity normalization reduced the number of differentially enriched peaks in the examined G4-targeted CUT&Tag libraries when compared to the appropriate matched untargeted CUT&Tag libraries. In fact, the number of identified G4s using this differential enrichment method was miniscule, suggesting that this colocalization of untargeted CUT&Tag reads and G4-targeted CUT&Tag reads makes this method of G4 identification unusable in the case of low-complexity untargeted CUT&Tag libraries. Increased sequencing depth may not alleviate this problem, given the expected low DNA yields and high duplication rates expected from untargeted CUT&Tag reactions, which would limit theoretical maximum library complexity.

Stringent optimization of experimental conditions will ideally eliminate untargeted tagmentation, resulting in untargeted libraries with very few detectable peaks, as in SRR14879749 [43]. Titration of salt concentrations to reduce untargeted tagmentation (as in [47]) can be performed for each cell line to reduce negative control DNA yields prior to sequencing, with a theoretically optimal untargeted CUT&Tag experiment yielding no measurable quantities of tagmented DNA under stringent high-salt conditions. However, if possible, sequencing of the untargeted controls would define any regions of the genome in the particular cell line of interest that may suffer from excessive untargeted tagmentation to enable detection of any residual untargeted tagmentation in G4-targeted CUT&Tag libraries.

Alternatively, to increase confidence in results from G4-targeted CUT&Tag experiments at a genome-wide scale, biological validation such as that used for G4Access [41] can be utilized. In this manuscript, a comparison of G4Access signals was performed following knockdown of the G4-resolving helicase DHX36 or using an untargeted control knockdown. In the control, various G4Access peaks are present at the *SIN3B* promoter with similar magnitudes of signal intensity. However, following DHX36 knockdown, one of the peaks substantially increases in magnitude above the nearby peaks [41], suggesting that it is a *bone fide* G4. However, the three other nearby peaks in G4Access signal do not share this increase in magnitude following DHX36 knockdown [41], suggesting that a certain amount of G4Access signal could also be reflective of local chromatin accessibility. This indicates that G4Access signal may also be somewhat biased for mapping of open chromatin, which is not surprising given the use of accessible chromatin as the substrate for MNase digestion. Inclusion of this targeted knockdown control provides increased confidence of a *bona fide* G4 at specific loci with the observed amplitude increase. Similarly, stabilization of G4s using PDS has led to increases in BG4 CUT&Tag signal amplitude compared to a DMSO control, although with low fold changes in signal amplitudes [43]. This suggests that any observed increase in G4-targeted CUT&Tag signal following G4 resolvase knockdown or G4 stabilization is reflective of G4 formation at a locus as opposed to being reflective of local chromatin accessibility.

However, these biological validation methods also carry caveats, given that DHX36 preferentially binds parallel G4s [18]. DHX36 and FANCJ (which unwinds G4s independent of topology [94, 95]) cooperate to resolve replisome-associated G4s [24], so double knockdown of both helicases (as performed previously [96]) could additionally serve as a good biological validation tool. Knockdown of other G4-topology-independent G4 resolvases such as PIF1 [97, 98] could also be utilized to potentially enhance G4 stabilization for CUT&Tag analysis, but this may increase stabilization of highly transient G4s. Additionally, use of PDS treatment prior to G4-targeted CUT&Tag analyses could also stabilize transient G4s [99], impacting which G4s are detected by G4-targeted CUT&Tag. Either thorough experimental optimization or inclusion of controls for biological validation could be used in G4-targeted CUT&Tag assays to define the landscape of G4s with high confidence.

## Supplementary Material

gkaf678_Supplemental_File

## Data Availability

All raw read data utilized for this analysis are available from GEO [53] and SRA [54], at the accessions included in Supplementary Tables S1, S4, S7, and S9. Peaks of G4s predicted by pqsfinder [86] mapped to the hg38 assembly of the human genome are available at https://pqsfinder.fi.muni.cz/genomes.
