# Supplementary material for: Untargeted CUT&Tag reads are enriched at accessible chromatin and restrict identification of potential G4-forming sequences in G4-targeted CUT&Tag experiments
Source: Nucleic Acids Res. 2025 Jul 19;53(14):gkaf678. doi: 10.1093/nar/gkaf678 (PMC12276005; doi:10.1093/nar/gkaf678)
Supplement: gkaf678_Supplemental_File [file gkaf678_supplemental_file.pdf]

Supplementary Information for:

Untargeted CUT&Tag reads are enriched at accessible chromatin and restrict identification of potential G4-forming sequences in G4-targeted CUT&Tag experiments

Matthew D. Thompson and Alicia K. Byrd

Supplementary Table 1. Untargeted CUT&Tag datasets acquired from GEO (1) used in Figures 1, 2, and Supplementary Figures 1 and 2.

Cell Type	Antibody	GEO Accession	SRA Accession	Source Publication
Kasumi-1	Non-targeting IgG	GSM5504185	SRR15353246	(2)
J-Lat 10.6	Non-targeting IgG	GSM6590675	SRR21625457	(3)
PC-3	Non-targeting IgG	GSM6919287	SRR22990288	(4)
H1	None	GSM6627988	SRR21859393	(5)
HEK293T	Non-targeting IgG	GSM6504650	SRR21211482	(6)
H1	Non-targeting IgG	GSM3560256	SRR8435038	(7)
Lung lymphatic endothelial cells	Non-targeting IgG	GSM6806707	SRR22577939	(8)
Karpas422	Non-targeting IgG	GSM5558304	SRR15729258	(9)
FHs74Int	Non-targeting IgG	GSM6574214	SRR21661660	(10)
Human umbilical cord mesenchymal stem cells	Non-targeting IgG	GSM7070802	SRR23641484	(11)
HepG2	Non-targeting IgG	GSM6306788	SRR20020486	(12)
HEK293T	Non-targeting IgG	GSM6267563	SRR19859937	(13)
THP-1	Non-targeting IgG	GSM5504188	SRR15353249	(2)
MOLM13	Non-targeting IgG	GSM7101045	SRR23876011	(14)
Cal27	Non-targeting IgG	GSM5641128	SRR16502256	(15)
HCT-116	Non-targeting IgG	GSM6235290	SRR19631169	(16)
Farage	Non-targeting IgG	GSM5558296	SRR15729250	(9)
U2OS	None	GSM5501191	SRR15337933	(17)
MCF7	None	GSM5501227	SRR15337969	(17)
K562	Non-targeting IgG	GSM3560264	SRR8435051	(7)
K562	None	GSM5501188	SRR15337930	(17)
HEK293T	Non-targeting IgG	GSM5395700	SRR14879749	(18)

Supplementary Table 2. Library size, complexity, and signal-to-noise statistics for each untargeted CUT&Tag dataset used in Figures 1 and 2 and Supplementary Figures 1 and 2. FRiP values were calculated using consensus intervals shared by at least 50% of datasets. Datasets SRR15353246, SRR21625457, SRR22990288, SRR21859393, and SRR23876011 were excluded from downsampling due to low numbers of unique reads, and remaining datasets were downsampled and then deduplicated to approximate the estimated number of unique reads (i.e. library complexity) in SRR15353249.

SRA Accession	Total reads (including duplicates)	# of peaks called	FRiP for consensus intervals (n=2,857)	Estimated number of unique reads	Estimated proportion of unique reads	Total reads after complexity normalization	# of peaks called after downsampling and deduplication	FRiP for consensus intervals (n=757) after downsampling
SRR15353246	4,749,644	105	0.02	28,252	0.01			
SRR21625457	244,832	1,210	0.02	53,529	0.22			
SRR22990288	4,711,111	683	0.03	71,691	0.02			
SRR21859393	24,877,558	2,137	0.02	265,073	0.01			
SRR21211482	28,662,521	225,658	0.00	14,426,740	0.50	915,173	21,833	0.00
SRR8435038	1,087,662	14,489	0.03	673,842	0.62	530,335	11,809	0.02
SRR22577939	20,393,886	33,998	0.01	2,875,907	0.14	1,231,076	27,471	0.01
SRR15729258	13,574,824	57,865	0.02	4,056,474	0.30	1,212,681	26,621	0.01
SRR21661660	4,996,073	64,977	0.01	3,815,105	0.76	602,090	13,825	0.01
SRR23641484	23,875,521	108,999	0.02	9,289,004	0.39	1,131,115	25,173	0.01
SRR20020486	21,215,484	172,177	0.00	16,396,178	0.77	612,199	14,592	0.00
SRR19859937	37,598,127	103,617	0.01	27,871,611	0.74	638,948	14,881	0.00
SRR15353249	9,935,804	4,331	0.05	477,382	0.05	189,466	4,319	0.03
SRR23876011	443,621	4,879	0.03	246,310	0.56			
SRR16502256	873,454	9,877	0.16	779,615	0.89	487,452	7,034	0.09
SRR19631169	1,221,014	13,897	0.04	746,476	0.61	487,755	11,032	0.02
SRR15729250	6,888,100	19,686	0.04	1,426,843	0.21	790,596	16,867	0.03
SRR15337933	2,286,664	20,962	0.09	1,443,223	0.63	609,041	11,425	0.05
SRR15337969	4,001,195	23,516	0.10	1,740,787	0.44	790,899	13,794	0.05
SRR8435051	1,674,550	21,105	0.05	1,125,647	0.67	585,979	12,276	0.03
SRR15337930	2,721,570	23,728	0.12	1,773,767	0.65	619,381	11,177	0.06
SRR14879749	44,609,438	104,445	0.00	19,595,046	0.44	1,040,590	23,377	0.00

Supplementary Table 3. Sample preparation factors for each untargeted CUT&Tag dataset, as plotted in Supplementary Figures 1 and 2.

SRA Accession	Input Type	Antibody	Fixation	Input Cell Number	# of PCR cycles
SRR15353246	Cells	Abcam, ab172730	0.1%, 2 min.	100,000	Not specified
SRR21625457	Nuclei	Abcam, ab172730	None	100,000	13 cycles
SRR22990288	Cells	No exact specification	None	100,000	14 cycles
SRR21859393	Nuclei	No IgG	0.1%, 2 min.	200,000	Not specified
SRR21211482	Nuclei	No exact specification	None	1,000,000	Not specified
SRR8435038	Cells	Abcam, ab46540	None	100,000	14 cycles
SRR22577939	Cells	AC005, Abclonal	None	90,000	Not specified
SRR15729258	Cells	Cell Sig. Tech., #2729	None	100,000	13 cycles
SRR21661660	Cells	Millipore, 12-370	None	100,000	15 cycles
SRR23641484	Nuclei	Proteintech 30000-0-AP	None	10,000	12 cycles
SRR20020486	Cells	Cell Sig. Tech., #2729	None	50,000	14 cycles
SRR19859937	Nuclei	No exact specification	None	100,000	Not specified
SRR15353249	Cells	Abcam, ab172730	0.1%, 2 min.	100,000	Not specified
SRR23876011	Cells	EpiCypher, 13-0042k	None	100,000	14 cycles
SRR16502256	Cells	Cell Sig. Tech., #2729	None	100,000	13 cycles
SRR19631169	Cells	Cell Sig. Tech., #2729	0.3%, 2 min.	500,000	Not specified
SRR15729250	Cells	Cell Sig. Tech., #2729	None	100,000	13 cycles
SRR15337933	Cells	No IgG	0.1%, 2 min.	100,000	10 cycles
SRR15337969	Cells	No IgG	0.1%, 2 min.	50,000	10 cycles
SRR8435051	Cells	Abcam, ab46540	None	100,000	14 cycles
SRR15337930	Cells	No IgG	0.1%, 2 min.	100,000	10 cycles
SRR14879749	Cells	No exact specification	None	500,000	Not specified

Supplementary Table 4. Reference information for datasets used in Figure 4. All datasets in this table were generated in K562 cells.

Sample	GEO Sample	SRA Accession(s)	Source publication	Biological Replicate
UCT 1	GSM5501188	SRR15337930	(17)	1
(i.e. no IgG CUT&Tag)	GSM5501194	SRR15337936	(17)	2
	GSM5501200	SRR15337942	(17)	3
UCT 2	GSM3560264	SRR8435051	(7)	1
(i.e. IgG CUT&Tag)	GSM3560264	SRR8435052	(7)	2
UCT 3	GSM5974972	SRR18503645	(19)	1
(i.e. IgG CUT&Tag)	GSM5974954	SRR18503663	(19)	2
	GSM5974936	SRR18503707	(19)	3
ATAC-seq	GSM5214756	SRR14105192, SRR14105193, SRR14105194, SRR14105195	(20)	1
ATAC-seq	GSM5214757	SRR14105196, SRR14105197, SRR14105198, SRR14105199	(20)	2
ATAC-seq	GSM5214758	SRR14105200, SRR14105201, SRR14105202, SRR14105203	(20)	3

Supplementary Table 5. Library sizes and background level statistics from K562-derived untargeted CUT&Tag datasets and ATAC-seq datasets. Reads from multiple sequencing runs were combined prior to downsampling to match the lowest complexity library and peak calling. Peaks for untargeted CUT&Tag datasets were called using SEACR (0.05 threshold), and peaks for ATAC-seq libraries were called using MACS2 (q-value = 0.05). Fraction of reads in peaks (FRiP) scores correspond to fraction of reads in all peaks across all samples. Consensus peaks within replicates were present in the indicated number (n) of biological replicates.

Sample	Biological Replicate	Total reads (including duplicates)	Estimated # of unique reads	Proportion of unique reads	# of reads (downsampled and deduplicated)	# of peaks called	FRiP	# of consensus peaks
UCT 1	1	2,721,554	1,773,746	0.65	840,247	14,435	0.36	4,555 (n = 3)
	2	3,214,808	1,670,172	0.52	904,555	15,385	0.36	
	3	2,199,680	1,014,528	0.46	736,014	13,255	0.35	
UCT 2	1	1,674,534	1,125,712	0.67	787,495	15,867	0.25	4,673
	2	1,056,158	695,008	0.66	682,563	13,649	0.26	(n = 2)
UCT 3	1	6,062,520	4,880,082	0.80	830,586	18,859	0.16	1,326 (n =3)
	2	4,237,598	902,404	0.21	828,744	17,794	0.24	
	3	6,431,934	4,460,840	0.69	929,212	19,949	0.21	
ATAC-seq	1	225,578,894	201,472,310	0.89	777,303	13,135	0.31	3,919 (n = 3)
	2	157,959,458	143,627,112	0.91	763,153	12,968	0.32	
	3	146,478,612	133,928,590	0.91	759,411	12,919	0.32	

Supplementary Table 6. Similarity metrics of replicate-conserved peaks called from untargeted CUT&Tag datasets from untreated K562 cells when compared to triplicate-replicated peaks (3,919 peaks) from ATAC-seq datasets from untreated K562 cells.

		Peak Calling		Precision/Recall Analysis			Jaccard Index			Fisher's Exact Test	
Target Dataset	Reference Dataset	SEACR Threshold	# of called peaks	Precision	Recall	F1-score	# of overlapping base pairs	# of non-overlapping base pairs	Jaccard Index	Odds ratio	P-value (one-tailed)
UCT 1	ATAC-seq	0.001	97	0.887	0.024	0.047	55,312	1,470,695	0.038	25,710.17	P<0.0001
consensus	peaks	0.01	1,236	0.673	0.222	0.334	353,975	1,978,826	0.179	22,13.38	P<0.0001
peaks		0.05	4,555	0.431	0.499	0.463	645,833	3,002,931	0.215	1,504.87	P<0.0001
(n=3)		0.1	6,426	0.358	0.575	0.441	710,954	3,432,556	0.207	1,448.81	P<0.0001
Shuffled	ATAC-seq	0.001	97	0.000	0.000	0.000	0	1,526,007	0.000	0.00	P=1
UCT 1	peaks	0.01	1,236	0.002	0.001	0.001	309	2,332,492	0.000	1.73	P=0.25
consensus		0.05	4,555	0.001	0.001	0.001	206	3,648,558	0.000	0.81	P=0.726
peaks		0.1	6,426	0.001	0.001	0.001	374	4,143,136	0.000	0.62	P=0.88
UCT 2	ATAC-seq	0.001	138	0.790	0.032	0.062	72,232	1,536,808	0.047	4,856.33	P<0.0001
consensus	peaks	0.01	1,395	0.576	0.218	0.316	348,598	2,253,126	0.155	1,331.16	P<0.0001
peaks		0.05	4,673	0.351	0.422	0.383	563,090	3,537,499	0.159	852.63	P<0.0001
(n=2)		0.1	6,692	0.282	0.476	0.354	613,649	4,176,903	0.147	741.54	P<0.0001
Shuffled	ATAC-seq	0.001	138	0.000	0.000	0.000	0	1,609,040	0.000	0.00	P=1
UCT 2	peaks	0.01	1,395	0.002	0.001	0.001	358	2,601,366	0.000	1.40	P=0.363
consensus		0.05	4,673	0.001	0.001	0.001	302	4,100,287	0.000	0.54	P=0.916
peaks		0.1	6,692	0.001	0.002	0.001	1,281	4,789,271	0.000	0.81	P=0.744
UCT 3	ATAC-seq	0.001	60	0.867	0.013	0.026	17,668	1,424,668	0.012	6,573.33	P<0.0001
consensus	peaks	0.01	445	0.634	0.066	0.119	62,733	1,467,117	0.043	2,376.92	P<0.0001
peaks		0.05	1,326	0.496	0.142	0.221	107,706	1,538,892	0.070	1,736.38	P<0.0001
(n=3)		0.1	1,978	0.442	0.184	0.260	131,982	1,591,727	0.083	1,550.39	P<0.0001
Shuffled	ATAC-seq	0.001	60	0.000	0.000	0.000	0	1,442,336	0.000	0.00	P=1
UCT 3	peaks	0.01	445	0.000	0.000	0.000	0	1,529,850	0.000	0.00	P=1
consensus		0.05	1,326	0.002	0.001	0.001	154	1,646,444	0.000	2.22	P=0.229
peaks		0.1	1,978	0.001	0.000	0.000	83	1,723,626	0.000	0.77	P=0.728

Supplementary Table 7. Reference information for datasets used in Figures 5 and 6 and Supplementary Figure 3. All datasets in this table were generated from K562 cells. Technical replicates are indicated in parentheses.

Sample	GEO Sample	SRA Accession(s)	Source publication	Biological Replicate
ATAC-seq	GSM5214756	SRR14105192, SRR14105193, SRR14105194, SRR14105195	(20)	1
ATAC-seq	GSM5214757	SRR14105196, SRR14105197, SRR14105198, SRR14105199	(20)	2
ATAC-seq	GSM5214758	SRR14105200, SRR14105201, SRR14105202, SRR14105203	(20)	3
BG4 CUT&Tag	GSM5501186 (T1), GSM5501187 (T2)	SRR15337928 (T1), SRR15337929 (T2)	(17)	1
BG4 CUT&Tag	GSM5501192 (T1), GSM5501193 (T2)	SRR15337934 (T1), SRR15337935 (T2)	(17)	2
BG4 CUT&Tag	GSM5501198 (T1), GSM5501199 (T2)	SRR15337940 (T1), SRR15337941 (T2)	(17)	3
Untargeted CUT&Tag Control (No IgG; i.e. UCT 1 R1)	GSM5501188	SRR15337930	(17)	1
Untargeted CUT&Tag Control (No IgG; UCT 1 R2)	GSM5501194	SRR15337936	(17)	2
Untargeted CUT&Tag Control (No IgG; UCT 1 R3)	GSM5501200	SRR15337942	(17)	3
Untargeted CUT&Tag Control (Non-targeting IgG; i.e. UCT 2 R1)	GSM3560264	SRR8435051	(7)	1
Untargeted CUT&Tag Control (Non-targeting IgG; i.e. UCT 2 R2)	GSM3560264	SRR8435052	(7)	2
Untargeted CUT&Tag Control (Non-targeting IgG; i.e. UCT 3 R1)	GSM5974972	SRR18503645	(19)	1
Untargeted CUT&Tag Control (Non-targeting IgG; i.e. UCT 3 R2)	GSM5974954	SRR18503663	(19)	2
Untargeted CUT&Tag Control (Non-targeting IgG; i.e. UCT 3 R1)	GSM5974936	SRR18503707	(19)	3
BG4 ChIP-seq	GSM4948705 (T1), GSM4948705 (T1), GSM4948706 (T2), GSM4948706 (T2), GSM4948707 (T3), GSM4948707 (T3)	SRR13161747 (T1), SRR13161748 (T1), SRR13161749 (T2), SRR13161750 (T2), SRR13161751 (T3), SRR13161752 (T3)	(21)	1
BG4 ChIP-seq	GSM4948708 (T1), GSM4948708 (T1), GSM4948709 (T2), GSM4948709 (T2), GSM4948710 (T3), GSM4948710 (T3)	SRR13161753 (T1), SRR13161754 (T1), SRR13161755 (T2), SRR13161756 (T2), SRR13161757 (T3), SRR13161758 (T3)	(21)	2
BG4 ChIP-seq	GSM4948711 (T1), GSM4948711 (T1), GSM4948712 (T2), GSM4948712 (T2), GSM4948713 (T3), GSM4948713 (T3)	SRR13161759 (T1), SRR13161760 (T1), SRR13161761 (T2), SRR13161762 (T2), SRR13161763 (T3), SRR13161763 (T3)	(21)	3
Input (BG4 ChIP-seq)	GSM4948764	SRR13161615, SRR13161616	(21)	1
Input (BG4 ChIP-seq)	GSM4948765	SRR13161617, SRR13161618	(21)	2
Input (BG4 ChIP-seq)	GSM4948766	SRR13161619, SRR13161620	(21)	3
G4Access	GSM5665726 (T1), GSM5665727 (T2), GSM5665728 (T3)	SRR16700952 (T1), SRR16700953 (T2), SRR16700954 (T3)	(22)	1
G4Access	GSM5665729	SRR16700955 (T1)	(22)	2
Input (G4Access)	GSM5665730	SRR16700956	(22)	1

Supplementary Table 7 continued

Sample	GEO Sample	SRA Accession(s)	Source publication	Biological Replicate
Chem-map (biotinylated PDS)	GSM6387151 (T1),<br>GSM6387152 (T2),<br>GSM6387153 (T3),<br>GSM6387154 (T4),<br>GSM6387155 (T5)	SRR20652907 (T1),<br>SRR20652906 (T2),<br>SRR20652905 (T3),<br>SRR20652904 (T4),<br>SRR20652903 (T5)	(23)	1
Chem-map (biotinylated PDS)	GSM6387156 (T1),<br>GSM6387157 (T2),<br>GSM6387158 (T3),<br>GSM6387159 (T4),<br>GSM6387160 (T5)	SRR20652902 (T1),<br>SRR20652901 (T2),<br>SRR20652900 (T3),<br>SRR20652899 (T4),<br>SRR20652898 (T5)	(23)	2
Chem-map (biotinylated PhenDC3)	GSM6387161 (T1),<br>GSM6387162 (T2),<br>GSM6387163 (T3),<br>GSM6387164 (T4),<br>GSM6387165 (T5)	SRR20652897 (T1),<br>SRR20652896 (T2),<br>SRR20652895 (T3),<br>SRR20652894 (T4),<br>SRR20652893 (T5)	(23)	1
Chem-map (biotinylated PhenDC3)	GSM6387166 (T1),<br>GSM6387167 (T2),<br>GSM6387168 (T3),<br>GSM6387169 (T4),<br>GSM6387170 (T5)	SRR20652892 (T1),<br>SRR20652891 (T2),<br>SRR20652890 (T3),<br>SRR20652889 (T4),<br>SRR20652888 (T5)	(23)	2
Chem-map (biotin control)	GSM6387231(T1),<br>GSM6387232 (T2),<br>GSM6387233 (T3),<br>GSM6387234 (T4),<br>GSM6387235 (T5)	SRR20652932 (T1),<br>SRR20652931 (T2),<br>SRR20652930 (T3),<br>SRR20652871 (T4),<br>SRR20652869 (T5)	(23)	1
Chem-map (biotin control)	GSM6387236 (T1),<br>GSM6387237 (T2),<br>GSM6387238 (T3),<br>GSM6387239 (T4),<br>GSM6387240 (T5)	SRR20652868 (T1),<br>SRR20652867 (T2),<br>SRR20652866 (T3),<br>SRR20652865 (T4),<br>SRR20652864 (T5)	(23)	2
SG4 ChIP-seq	GSM6297320 (T1),<br>GSM6297321 (T2),<br>GSM6297322 (T3)	SRR19997617 (T1),<br>SRR19997616 (T2),<br>SRR19997615 (T3)	(24)	1
SG4 ChIP-seq	GSM6297323 (T1),<br>GSM6297324 (T2),<br>GSM6297325 (T3)	SRR19997614 (T1),<br>SRR19997613 (T2),<br>SRR19997612 (T3)	(24)	2
SG4 ChIP-seq	GSM6297326 (T1),<br>GSM6297327 (T2),<br>GSM6297328 (T3)	SRR19997611 (T1),<br>SRR19997610 (T2),<br>SRR19997609 (T3)	(24)	3
Input (SG4 ChIP-seq)	GSM6297329	SRR19997608	(24)	1
Input (SG4 ChIP-seq)	GSM6297330	SRR19997607	(24)	2
Input (SG4 ChIP-seq)	GSM6297331	SRR19997606	(24)	3
pqsfinder (v2.0.1), hg38 track	N/A	N/A	(25)	N/A
G4-seq, K^+ stabilized<br>(hg19 signal regions lifted over to hg38 with UCSC liftover (26, 27) and strands were merged)	GSM3003539	N/A	(28)	N/A

Supplementary Table 8. Library sizes and background level statistics from K562-derived untargeted CUT&Tag datasets in Figures 5 and 6, and Supplementary Figure 3. Tn5-derived libraries (i.e. CUT&Tag, Chem-map, ATAC-seq) were downsampled to match the Untargeted CUT&Tag 2 replicate 2 library (UCT 2 R2) with the lowest number of unique reads, except for Chem-map Biotin Control Biological Replicate 2 which had a further limiting number of reads. Reads from ChIP-seq- and G4Access-derived libraries were not downsampled. Reads for all libraries were then deduplicated, giving the number of unique reads. Peak calling was performed on the unique reads using a threshold of 0.05 for SEACR (top 5% of signal blocks) without a negative control for CUT&Tag-derived datasets or using an FDR threshold of 0.05 with MACS3 and input controls for other datasets (i.e. ChIP-seq and G4Access). Peaks for ATAC-seq libraries were called using an FDR threshold of 0.05 with MACS3 without an input control. Fraction of reads in peaks (FRiP) scores correspond to fraction of reads in all peaks across all samples. Consensus peaks within replicates were present in the indicated number (n) of biological replicates.

Sample	Biological Replicate	Total reads (including duplicates)	Estimated # of unique reads	Proportion of unique reads	# of reads (deduplicated with Tn5-derived datasets downsampled)	# of peaks called	FRiP	# of consensus peaks
ATAC-seq	1	146,478,612	133,931,878	0.91	777,303	13,135	0.31	3,919 (n = 3)
ATAC-seq	2	157,959,458	143,627,180	0.91	763,153	12,968	0.32	
ATAC-seq	3	225,578,894	201,475,094	0.89	759,411	12,919	0.32	
BG4 CUT&Tag	1	18,241,466	14,144,940	0.78	878,943	3,198	0.31	1,770 (n = 3)
BG4 CUT&Tag	2	30,263,588	23,716,452	0.78	877,747	3,395	0.26	
BG4 CUT&Tag	3	33,772,338	25,632,040	0.76	905,549	3,489	0.27	
Untargeted CUT&Tag (No IgG; i.e. UCT 1 R1)	1	2,721,554	1,773,746	0.65	840,247	2,946	0.31	4,555 (n = 3)
Untargeted CUT&Tag (No IgG; i.e. UCT 1 R2)	2	3,214,808	1,670,172	0.52	904,555	3,134	0.30	
Untargeted CUT&Tag (No IgG; i.e. UCT 1 R3)	3	2,199,680	1,014,528	0.46	736,014	2,699	0.29	
Untargeted CUT&Tag (Non-targeting IgG; i.e. UCT 2 R1)	1	1,674,534	1,125,712	0.67	787,495	3,182	0.17	4,673 (n = 2)
Untargeted CUT&Tag (Non-targeting IgG; i.e. UCT 2 R2)	2	1,056,158	695,008	0.66	682,563	2,752	0.19	
Untargeted CUT&Tag (Non-targeting IgG; i.e. UCT 3 R1)	1	6,062,520	4,880,082	0.80	830,586	3,762	0.09	1,326 (n =3)
Untargeted CUT&Tag (Non-targeting IgG; i.e. UCT 3 R2)	2	4,237,598	902,404	0.21	828,744	3,555	0.20	
Untargeted CUT&Tag (Non-targeting IgG; i.e. UCT 3 R3)	3	6,431,934	4,460,840	0.69	929,212	4,016	0.15	
Chem-map (biotinylated PDS)	1	85,143,870	60,167,014	0.71	975,997	3,929	0.21	1,779 (n = 2)
Chem-map (biotinylated PDS)	2	48,255,964	37,183,128	0.77	894,344	3,778	0.17	
Chem-map (biotinylated PhenDC3)	1	70,259,612	54,540,140	0.78	892,256	3,711	0.19	1,719 (n = 2)
Chem-map (biotinylated PhenDC3)	2	14,005,086	11,496,506	0.82	835,408	3,275	0.25	
Chem-map (biotin control)	1	28,976,504	10,398,606	0.36	1,718,595	7, 359	0.09	1,436 (n = 2)
Chem-map (biotin control)	2				860,777	2,448	0.23	
BG4 ChIP-seq	1				94,234,753	10,024	0.05	7,777 (n=3)
BG4 ChIP-seq	2				96,270,093	11,945	0.06	
BG4 ChIP-seq	3				107,810,633	13,452	0.06	
Input (BG4 ChIP-seq)	1				40,085,639		0.02	
G4Access	1				11,368,460	56,500	0.23	24,602 (n=2)
G4Access	2				127,643,594	35,119	0.06	
Input (G4Access)	1				77,628,846		0.02	
SG4 ChIP-seq	1				104,008,803	3,641	0.04	4,634 (n=2)
SG4 ChIP-seq	2				102,190,159	1,005	0.04	
SG4 ChIP-seq	3				76,110,444	5,161	0.04	
Input (SG4 ChIP-seq)	1				6,944,002		0.02	

Supplementary Table 9. Dataset reference information for Supplementary Figure 4. All datasets in this table were generated from HEK293T cells. BG4 CUT&Tag libraries from different sources are distinguished with superscripts (*ref. (18), ^ref. (29), #ref. (30)). Technical replicates are indicated in parentheses.

Sample	GEO Sample	SRA Accession(s)	Source Publication	Biological Replicate
ATAC-seq	GSM7493124	SRR24927146	(31)	1
ATAC-seq	GSM7493125	SRR24927145	(31)	2
Omni-ATAC-seq	GSM5395701	SRR14879750	(18)	1
BG4 CUT&Tag*	GSM5395699	SRR14879748	(18)	1
BG4 CUT&Tag*	GSM5401689	SRR14917105	(18)	2
IgG CUT&Tag	GSM5395700	SRR14879749	(18)	1
BG4 CUT&Tag, DMSO control*	GSM5395733	SRR14879782	(18)	1
BG4 CUT&Tag, PDS treatment*	GSM5395734	SRR14879783	(18)	1
BG4 CUT&Tag^	GSM7815665 (T1), GSM7815666 (T2)	SRR26244912 (T1), SRR26244911 (T2)	(29)	1
BG4 CUT&Tag^	GSM7815667 (T1), GSM7815667 (T2)	SRR26244910 (T1), SRR26244909 (T2)	(29)	2
BG4 CUT&Tag^	GSM7815669 (T1), GSM7815670 (T2)	SRR26244908 (T1), SRR26244907 (T2)	(29)	3
SG4 CUT&Tag	GSM7815849 (T1), GSM7815850 (T2)	SRR26244733 (T1), SRR26244732 (T2)	(29)	1
SG4 CUT&Tag	GSM7815851 (T1), GSM7815852 (T2)	SRR26244731 (T1), SRR26244730 (T2)	(29)	2
SG4 CUT&Tag	GSM7815853	SRR26244729	(29)	3
SG4 R105A CUT&Tag	GSM7815843	SRR26244739	(29)	1
SG4 R105A CUT&Tag	GSM7815844	SRR26244738	(29)	2
Chem-map (biotinylated PDS)	GSM7815775 (T1), GSM7815776 (T2)	SRR26244802 (T1), SRR26244801 (T2)	(29)	1
Chem-map (biotinylated PDS)	GSM7815777 (T1), GSM7815778 (T2)	SRR26244800 (T1), SRR26244799 (T2)	(29)	2
Chem-map (biotinylated PDS)	GSM7815779 (T1), GSM7815780 (T2)	SRR26244798 (T1), SRR26244797 (T2)	(29)	3
BG4 CUT&Tag#	GSM7507860	SRR25010686	(30)	1
BG4 CUT&Tag#	GSM7507861	SRR25010685	(30)	2
BG4 CUT&Tag#	GSM7507862	SRR25010684	(30)	3
iMotif CUT&Tag	GSM7507863	SRR25010683	(30)	1
iMotif CUT&Tag	GSM7507864	SRR25010682	(30)	2
iMotif CUT&Tag	GSM7507865	SRR25010681	(30)	3
G4P ChIP-seq	GSM3907020	SRR9603961	(32)	1
G4P ChIP-seq	GSM3907022	SRR9603963	(32)	2
G4P ChIP-seq Input	GSM3907021	SRR9603962	(32)	1
G4P ChIP-seq Input	GSM3907023	SRR9603964	(32)	2

Supplementary Table 10. Library sizes and background level statistics from HEK293T-derived untargeted CUT&Tag datasets in Supplementary Figures 4 and 5, and SG4 CUT&Tag in Figures 7 and 8, and Supplementary Figure 6. Tn5-derived libraries (i.e. CUT&Tag, Chem-map, ATAC-seq, Omni-ATAC-seq) were downsampled to match the SG4 R105A CUT&Tag Control Replicate 2 dataset with the limiting number of unique reads. Reads from ChIP-seq-derived libraries were not downsampled. Reads for all libraries were then deduplicated, giving the number of unique reads. Peak calling was performed using the unique reads with a threshold of 0.05 for SEACR (top 5% of signal blocks) without a negative control for Tn5-derived datasets or using an FDR threshold of 0.05 with MACS3 and input controls for non-Tn5-derived datasets (i.e. ChIP-seq). Fraction of reads in peaks (FRiP) scores correspond to fraction of reads in all peaks across all samples. Consensus peaks within replicates were present in the indicated number (n) of biological replicates. BG4 CUT&Tag libraries from different sources are distinguished with superscripts ([*]ref. (18), [^]ref. (29), [#]ref. (30)).

Sample	Biological Replicate	Total reads (including duplicates)	Estimated # of unique reads	Proportion of unique reads	# of reads (deduplicated with Tn5-derived datasets downsampled)	# of peaks called	FRiP	# of consensus peaks
ATAC-seq	1	40,227,158	34,173,920	0.85	708,809	9,409	0.41	3,277
ATAC-seq	2	43,373,014	36,850,712	0.85	710,323	9,145	0.41	(n = 2)
Omni-ATAC-seq	1	12,573,110	5,084,222	0.40	1,156,587	13,202	0.98	
BG4 CUT&Tag[*]	1	55,574,056	41,644,414	0.75	804,398	16,578	0.35	2,358
BG4 CUT&Tag[*]	2	129,002,214	99,807,403	0.77	777,474	18,032	0.33	(n=2)
IgG CUT&Tag[*]	1	44,609,506	19,596,615	0.44	1,304,052	28,593	0.23	
BG4 CUT&Tag, DMSO control	1	8,240,454	6,543,109	0.79	740,999	14,837	0.53	
BG4 CUT&Tag, PDS treatment	1	12,137,508	9,164,116	0.76	783,457	14,888	0.57	
BG4 CUT&Tag[^]	1	49,209,748	39,434,249	0.80	751,573	14,689	0.46	4,089 (n=3)
BG4 CUT&Tag[^]	2	53,536,012	39,651,801	0.74	810,319	16,792	0.39	
BG4 CUT&Tag[^]	3	52,578,182	41,690,589	0.79	760,092	15,703	0.40	
SG4 CUT&Tag	1	23,464,846	7,844,989	0.33	1,571,014	29,290	0.43	8,832 (n=3)
SG4 CUT&Tag	2	28,826,140	10,588,228	0.37	1,501,598	25,643	0.51	
SG4 CUT&Tag	3	13,846,210	5,169,104	0.37	1,357,094	22,003	0.54	
SG4 R105A CUT&Tag	1	2,201,600	891,147	0.40	711,214	13,670	0.47	2,865
SG4 R105A CUT&Tag	2	1,487,786	605,050	0.41	559,613	12,344	0.35	(n=2)
Chem-map (biotinylated PDS)	1	27,194,264	16,216,518	0.60	980,671	19,412	0.42	4,350 (n=3)
Chem-map (biotinylated PDS)	2	29,082,164	16,828,948	0.58	1,011,784	21,162	0.37	
Chem-map (biotinylated PDS)	3	28,568,092	16,568,308	0.58	1,010,230	21,048	0.38	
BG4 CUT&Tag[#]	1	29,706,824	8,638,915	0.29	1,751,123	35,747	0.39	6,357 (n=3)
BG4 CUT&Tag[#]	2	35,331,622	15,664,362	0.44	1,295,254	25,066	0.44	
BG4 CUT&Tag[#]	3	49,746,410	20,209,101	0.41	1,425,754	28,519	0.37	
iMotif CUT&Tag	1	41,273,320	10,761,225	0.26	1,981,213	38,719	0.39	6,973 (n=3)
iMotif CUT&Tag	2	57,296,700	16,482,297	0.29	1,918,601	37,310	0.42	
iMotif CUT&Tag	3	56,878,672	14,349,055	0.25	2,116,762	41,949	0.37	
G4P ChIP-seq	1				97,820,698	33,293	0.23	13,582
G4P ChIP-seq	2				92,913,289	16,423	0.22	(n=2)
G4P ChIP-seq Input	1				184,505,051		0.16	
G4P ChIP-seq Input	2				97,683,648		0.18	

Supplementary Table 11. Similarity metrics of peaks (0.01 threshold, top 1% of signal blocks) called by SEACR from untargeted CUT&Tag datasets from untreated K562 cells when compared to consensus peaks from the indicated reference datasets, as plotted in Figure 6.

		Peak Calling	Precision/Recall Analysis			Jaccard Index			Fisher's Exact Test	
Target Dataset	Reference Dataset	# of called peaks	Precision	Recall	F1-score	Overlap (bp)	No overlap (bp)	Jaccard Index	Odds ratio	P-value
UCT 1 consensus peaks	ATAC-seq (3,919 peaks)	0.673	0.673	0.222	0.334	353,975	1978826	0.179	2213.38	P<0.0001
Shuffled UCT 1 consensus peaks		0.002	0.002	0.001	0.001	309	2332492	0.000	1.73	P=0.25
UCT 2 consensus peaks		0.576	0.576	0.218	0.316	348,598	2253126	0.155	1331.16	P<0.0001
Shuffled UCT 2 consensus peaks		0.002	0.002	0.001	0.001	358	2601366	0.000	1.40	P=0.36
UCT 3 consensus peaks		0.634	0.634	0.066	0.119	62,733	1467117	0.043	2376.92	P<0.0001
Shuffled UCT3 consensus peaks		0.000	0.000	0.000	0.000	0	1529850	0.000	0.00	P=1.00
UCT 1 consensus peaks	BG4 ChIP-seq (7,777 peaks)	0.887	0.887	0.176	0.294	304,327	1,892,068	0.161	Inf.	P<0.0001
BShuffled UCT 1 consensus peaks		0.002	0.002	0.000	0.000	437	2,195,958	0.000	0.71	P=0.77
UCT 2 consensus peaks		0.753	0.753	0.175	0.284	265,483	2,199,835	0.121	21513.35	P<0.0001
Shuffled UCT 2 consensus peaks		0.002	0.002	0.000	0.001	918	2,464,400	0.000	0.84	P=0.69
UCT 3 consensus peaks		0.888	0.888	0.052	0.098	81,109	1,312,335	0.062	14839.75	P<0.0001
Shuffled UCT3 consensus peaks		0.000	0.000	0.000	0.000	0	1,393,444	0.000	0.00	P=1.00
UCT 1 consensus peaks	BG4 CUT&Tag (1,770 peaks)	0.568	0.568	0.397	0.467	431,405	1,756,685	0.246	2642.08	P<0.0001
Shuffled UCT 1 consensus peaks		0.001	0.001	0.001	0.001	240	2,187,850	0.000	0.97	P=0.65
UCT 2 consensus peaks		0.430	0.430	0.342	0.381	377,702	2,079,311	0.182	1315.92	P<0.0001
Shuffled UCT 2 consensus peaks		0.002	0.002	0.002	0.002	808	2,456,205	0.000	2.40	P=0.13
UCT 3 consensus peaks		0.688	0.688	0.150	0.246	80,128	1,305,011	0.061	4761.48	P<0.0001
Shuffled UCT3 consensus peaks		0.000	0.000	0.000	0.000	0	1,385,139	0.000	0.00	P=1.00
UCT 1 consensus peaks	G4Access (24,602 peaks)	0.956	0.956	0.082	0.150	383,044	3,711,501	0.103	Inf.	P<0.0001
Shuffled UCT 1 consensus peaks		0.006	0.006	0.000	0.001	769	4,093,776	0.000	0.81	P=0.88
UCT 2 consensus peaks		0.875	0.875	0.086	0.156	341,165	4,022,303	0.085	Inf.	P<0.0001
Shuffled UCT 2 consensus peaks		0.006	0.006	0.000	0.001	1046	4,362,422	0.000	1.01	P=0.53
UCT 3 consensus peaks		0.892	0.892	0.019	0.036	79,417	3,212,177	0.025	Inf.	P<0.0001
Shuffled UCT3 consensus peaks		0.002	0.002	0.000	0.000	37	3,291,557	0.000	0.73	P=0.75
UCT 1 consensus peaks	G4-seq (427,833 peaks)	0.666	0.666	0.003	0.006	187,767	54,565,604	0.003	4147.94	P<0.0001
Shuffled UCT 1 consensus peaks		0.108	0.108	0.000	0.001	17,786	54,735,585	0.000	2.08	P=0.25
UCT 2 consensus peaks		0.693	0.693	0.004	0.007	261,978	54,760,316	0.005	2281.90	P<0.0001

Shuffled UCT 2 consensus peaks		0.116	0.116	0.000	0.001	21,987	55,000,307	0.000	1.71	P=0.33
UCT 3 consensus peaks		0.530	0.530	0.001	0.001	38,976	53,911,444	0.001	5226.16	P<0.0001
Shuffled UCT3 consensus peaks		0.052	0.052	0.000	0.000	2,496	53,947,924	0.000	4.53	P=0.20
UCT 1 consensus peaks	PDS Chem-map (1,779 peaks)	0.633	0.633	0.443	0.521	430,160	1,566,191	0.275	4369.83	P<0.0001
Shuffled UCT 1 consensus peaks		0.002	0.002	0.001	0.001	308	1,996,043	0.000	1.05	P=0.61
UCT 2 consensus peaks		0.513	0.513	0.411	0.456	404,912	1,860,362	0.218	2451.79	P<0.0001
Shuffled UCT 2 consensus peaks		0.001	0.001	0.001	0.001	896	2,264,378	0.000	1.73	P=0.32
UCT 3 consensus peaks		0.676	0.676	0.152	0.248	75,435	1,117,965	0.067	4963.92	P<0.0001
Shuffled UCT3 consensus peaks		0.002	0.002	0.001	0.001	41	1,193,359	0.000	0.00	P=1.00
UCT 1 consensus peaks	PhenDC3 Chem-map (1,719 peaks)	0.633	0.633	0.461	0.533	440,687	1,576,779	0.279	Inf.	P<0.0001
Shuffled UCT 1 consensus peaks		0.001	0.001	0.001	0.001	241	2,017,225	0.000	1.10	P=0.06
UCT 2 consensus peaks		0.518	0.518	0.432	0.471	419,937	1,866,452	0.225	Inf.	P<0.0001
Shuffled UCT 2 consensus peaks		0.001	0.001	0.001	0.001	700	2,285,689	0.000	0.99	P=0.57
UCT 3 consensus peaks		0.672	0.672	0.154	0.251	75,643	1,138,872	0.066	Inf.	P<0.0001
Shuffled UCT3 consensus peaks		0.000	0.000	0.000	0.000	0	1,214,515	0.000	1.26	P=0.04
UCT 1 consensus peaks	pqsfinder (1,345,937 peaks)	0.937	0.937	0.004	0.008	164,264	42,982,654	0.004	Inf.	P<0.0001
Shuffled UCT 1 consensus peaks		0.192	0.192	0.000	0.001	13,430	43,133,488	0.000	0.65	P=0.79
UCT 2 consensus peaks		0.934	0.934	0.005	0.010	213,481	43,202,360	0.005	2821.50	P<0.0001
Shuffled UCT 2 consensus peaks		0.196	0.196	0.000	0.001	16,275	43,399,566	0.000	2.04	P=0.14
UCT 3 consensus peaks		0.769	0.769	0.001	0.001	26,843	42,317,124	0.001	17096.20	P<0.0001
Shuffled UCT3 consensus peaks		0.101	0.101	0.000	0.000	1,979	42,341,988	0.000	0.00	P=1.00
UCT 1 consensus peaks	SG4 ChIP-seq (4,634 peaks)	0.497	0.497	0.285	0.362	119,166	1,205,894	0.099	2213.38	P<0.0001
Shuffled UCT 1 consensus peaks		0.001	0.001	0.000	0.000	52	1,325,008	0.000	1.73	P=0.25
UCT 2 consensus peaks		0.368	0.368	0.227	0.281	91,951	1,502,032	0.061	1331.16	P<0.0001
Shuffled UCT 2 consensus peaks		0.002	0.002	0.001	0.001	290	1,593,693	0.000	1.40	P=0.36
UCT 3 consensus peaks		0.452	0.452	0.085	0.143	33,504	488,605	0.069	2376.92	P<0.0001
Shuffled UCT3 consensus peaks		0.000	0.000	0.000	0.000	0	522,109	0.000	0.00	P=1.00

Supplementary Table 12. Peak calling and precision/recall statistics related to Figure 8B and Supplementary Figure 6. Differentially enriched/depleted peaks were called using SEACR with a 0.01 threshold without a negative control for both G4-mapping datasets and controls, prior to peak differential enrichment analysis using DEseq2 (33) within DiffBind (34). Other peaks were called from the target datasets using SEACR with the indicated settings, and BG4 ChIP-seq peaks called using MACS3 with a q-value cut-off of 0.05 and were conserved among all technical replicates of all biological replicates.

G4-mapping Dataset and Control	Peak calling method	# of peaks	Down-sampled?	Reference Dataset 1	Precision	Recall	Reference Dataset 2	Precision	Recall
BG4 CUT&Tag	Diff. Enriched	2,543	-	G4-seq	0.627	0.007	BG4	0.403	0.144
(n=3)	Diff. Depleted	6,136	-	(n=427,833)	0.801	0.046	ChIP-seq	0.259	0.269
and	SEACR, 0.001	2,329	-		0.852	0.011	(n=7,777)	0.912	0.351
IgG CUT&Tag	SEACR, 0.003	7,511	-		0.760	0.028		0.706	0.786
(n=3)	SEACR, 0.005	11,871	-		0.693	0.039		0.529	0.917
	SEACR, 0.007	15,470	-		0.641	0.045		0.421	0.948
	SEACR, 0.01	19,940	-		0.584	0.051		0.332	0.960
	SEACR w/ IgG, stringent	6,861	-		0.77	0.026		0.732	0.749
	SEACR w/ IgG, relaxed	10,978	-		0.698	0.036		0.558	0.896
	Diff. Enriched	1,008	+		0.771	0.004		0.706	0.102
	Diff. Depleted	1,227	+		0.896	0.016		0.47	0.110
	SEACR, 0.001	222	+		0.824	0.001		0.995	0.043
	SEACR, 0.003	758	+		0.765	0.002		0.958	0.122
	SEACR, 0.005	1,348	+		0.713	0.004		0.941	0.203
	SEACR, 0.007	1,916	+		0.681	0.005		0.925	0.276
	SEACR, 0.01	2,855	+		0.646	0.006		0.880	0.378
	SEACR w/ IgG, stringent	72	+		0.861	0.000		1	0.015
	SEACR w/ IgG, relaxed	455	+		0.798	0.001		0.982	0.080
SG4 CUT&Tag	SEACR, 0.001	1,114	-	G4-seq	0.913	0.007			
(n=3) and SG4	SEACR, 0.003	3,441	-	(n=427,833)	0.839	0.016			
R105A CUT&Tag	SEACR, 0.005	5,781	-		0.800	0.024			
(n=2)	SEACR, 0.007	8,050	-		0.770	0.031			
	SEACR, 0.01	10,981	-		0.728	0.038			
	SEACR w/ IgG, stringent	2,734	-		0.858	0.014			
	SEACR w/ IgG, relaxed	6,338	-		0.789	0.026			
	SEACR, 0.001	295	+		0.908	0.001			
	SEACR, 0.003	906	+		0.828	0.003			
	SEACR, 0.005	1,554	+		0.792	0.005			
	SEACR, 0.007	2,261	+		0.749	0.007			
	SEACR, 0.01	3,265	+		0.708	0.009			
	SEACR w/ IgG, stringent	193	+		0.927	0.001			
	SEACR w/ IgG, relaxed	753	+		0.829	0.003			
PDS Chem-map	SEACR, 0.001	2,655	-	G4-seq	0.935	0.037	BG4	0.737	0.367
(n=2) and Biotin	SEACR, 0.003	8,185	-	(n=427,833)	0.861	0.072	ChIP-seq	0.56	0.741
Chem-map (n=2)	SEACR, 0.005	13,210	-		0.809	0.096	(n=7,777)	0.422	0.874
	SEACR, 0.007	17,956	-		0.766	0.114		0.33	0.921
	SEACR, 0.01	24,562	-		0.728	0.128		0.271	0.943
	SEACR w/ IgG, stringent	610	-		0.987	0.006		0.894	0.044
	SEACR w/ IgG, relaxed	151	-		0.98	0.015		0.813	0.121
	SEACR, 0.001	304	+		0.799	0.001		0.947	0.053
	SEACR, 0.003	884	+		0.725	0.002		0.915	0.135
	SEACR, 0.005	1,514	+		0.67	0.004		0.864	0.205
	SEACR, 0.007	2,129	+		0.641	0.005		0.812	0.263
	SEACR, 0.01	2,916	+		0.611	0.006		0.757	0.327
	SEACR w/ IgG, stringent	0	+						
	SEACR w/ IgG, relaxed	0	+						

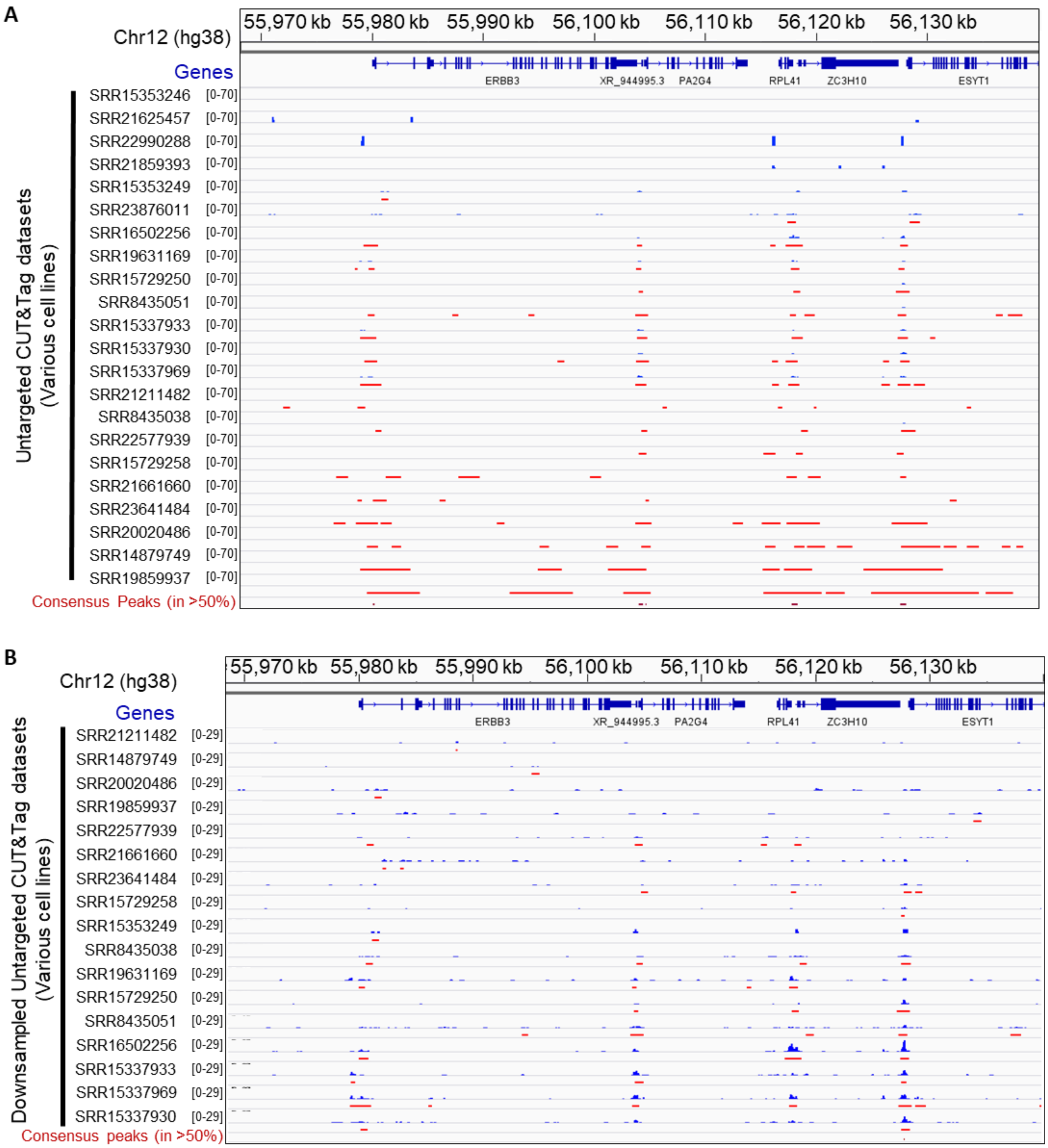

Supplementary Figure 1. Untargeted CUT&Tag datasets share loci of signal enrichment but with varying amplitudes. (A) Counts-per-million reads (CPM)-normalized read counts (blue) of deduplicated untargeted CUT&Tag libraries at a representative locus on chromosome 12, as in Figure 1B, but read counts are instead plotted on the same indicated axis range scaled to the same amplitude as determined by the library with the highest individual signal enrichment at this locus. SEACR-called peaks of signal enrichment (red) are plotted for each library, alongside a set of consensus peaks (maroon; n = 2,857), derived from genomic intervals present in at least 50% of the libraries. **(B)** CPM-normalized read counts of downsampled and deduplicated untargeted CUT&Tag libraries, as in Figure 2B, but read counts are instead plotted on the same indicated axis range scaled to the same amplitude as determined by the library with the highest individual signal enrichment at this locus.

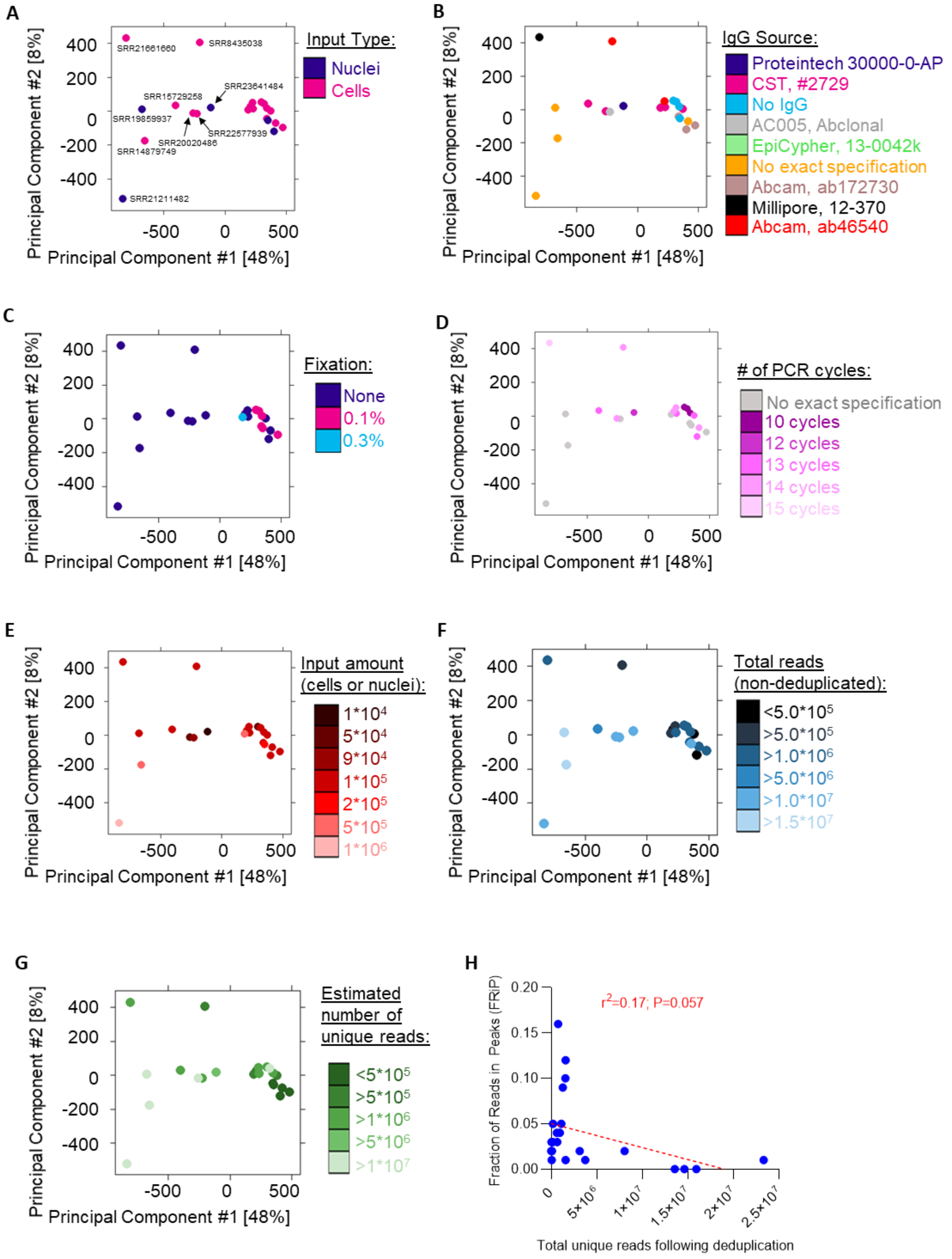

Supplementary Figure 2. Untargeted CUT&Tag library genome-wide read distributions are not strongly correlated with sample preparation factors. (A) Principle Component Analysis (PCA) of normalized read counts from deduplicated untargeted CUT&Tag libraries (from various cell lines) across any site of signal enrichment of each sample, as in Figure 1A. Datasets are labelled by input type (i.e. what was bound to concanavalin A beads prior to tagmentation during the associated CUT&Tag protocol. **(B)** PCA as in (A), but datasets are labelled by negative control antibody use and specification, where available. **(C)** PCA as in (A), but datasets are labelled by formaldehyde fixation prior to CUT&Tag. **(D)** PCA as in (A), but datasets are labelled by the number of PCR cycles used for PCR amplification and barcoding following CUT&Tag, where available. **(E)** PCA as in (A), but datasets are labelled by the number of cells or nuclei used for CUT&Tag. **(F)** PCA as in (A), but datasets are labelled by the total number of reads (non-deduplicated). **(G)** PCA as in (A), but datasets are labelled by the total number of unique reads (i.e. library complexity). **(H)** Scatterplot and line of best fit (r^2=0.17, P=0.057) of fraction of reads in peaks (FRiP) scores of each library at a set of consensus peaks (n = 2,857; see Figure 1B, bottom track) compared to total number of unique reads (i.e. library complexity).

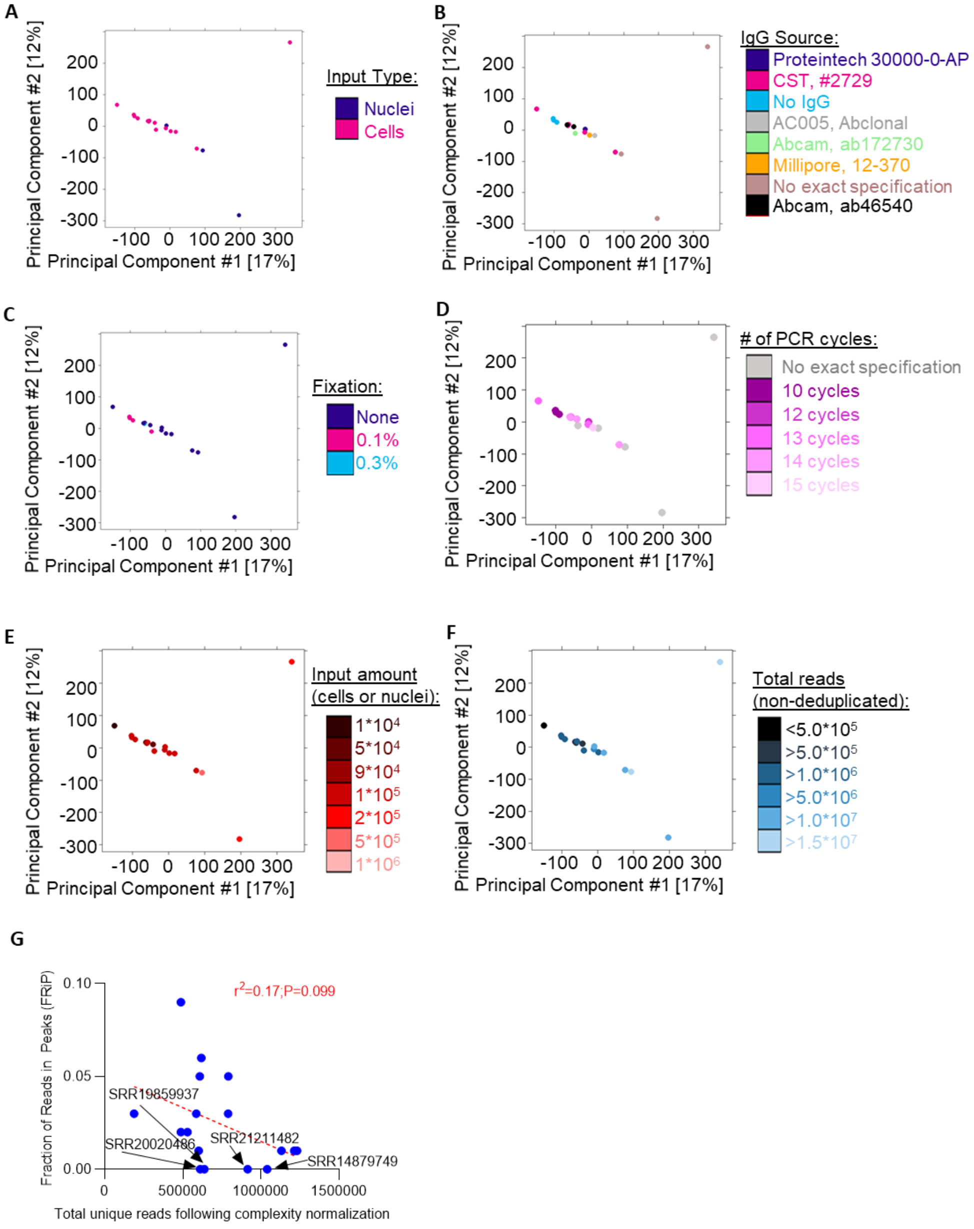

Supplementary Figure 3. Untargeted CUT&Tag library genome-wide read distributions are not strongly correlated with sample preparation factors following complexity normalization. (A) Principle Component Analysis (PCA) of normalized read counts from complexity-normalized (i.e. downsampled and deduplicated) untargeted CUT&Tag libraries (from various cell lines) across any site of signal enrichment of each sample, as in Figure 1A. Datasets are labelled by input type (i.e. what was bound to concanavalin A beads prior to tagmentation during the associated CUT&Tag protocol). **(B)** PCA as in (A), but datasets are labelled by negative control antibody use and specification, where available. **(C)** PCA as in (A), but datasets are labelled by formaldehyde fixation prior to CUT&Tag. **(D)** PCA as in (A), but datasets are labelled by the number of PCR cycles used for PCR amplification and barcoding following CUT&Tag, where available. **(E)** PCA as in (A), but datasets are labelled by the number of cells or nuclei used for CUT&Tag. **(F)** PCA as in (A), but datasets are labelled by the total number of unique reads (i.e. library complexity) prior to complexity normalization (i.e. downsampling and deduplication). **(G)** Scatterplot and line of best fit (r^2=0.17, P=0.099) of fraction of reads in peaks (FRiP) scores of each library at a set of consensus peaks (n = 2,857; see Figure 1B, bottom track) compared to total number of unique reads following complexity normalization (i.e. downsampling with deduplication).

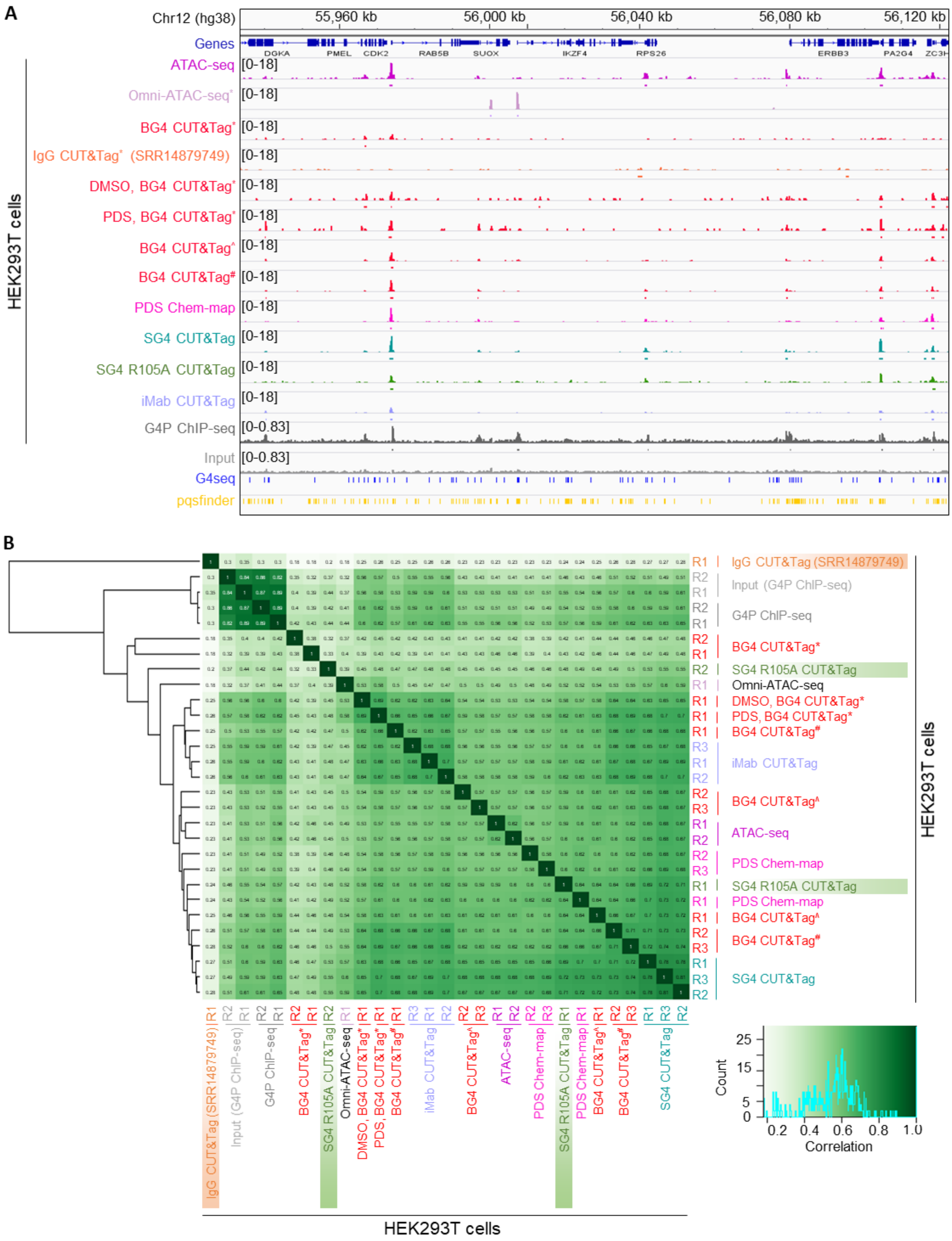
A
Chr12 (hg38)
55,960 kb
56,000 kb
56,040 kb
56,080 kb
56,120 kb
Genes
DGKA
PMEL
CDK2
RAB5B
SUOX
IKZF4
RPS26
ERBB3
PA2G4
ZC3H
HEK293T cells
ATAC-seq [0-18]
Omni-ATAC-seq* [0-18]
BG4 CUT&Tag* [0-18]
IgG CUT&Tag* (SRR14879749) [0-18]
DMSO, BG4 CUT&Tag* [0-18]
PDS, BG4 CUT&Tag* [0-18]
BG4 CUT&Tag^ [0-18]
BG4 CUT&Tag# [0-18]
PDS Chem-map [0-18]
SG4 CUT&Tag [0-18]
SG4 R105A CUT&Tag [0-18]
iMab CUT&Tag [0-18]
G4P ChIP-seq [0-0.83]
Input [0-0.83]
G4seq
pqsfinder
B
IgG CUT&Tag (SRR14879749)
Input (G4P ChIP-seq)
G4P ChIP-seq
BG4 CUT&Tag*
SG4 R105A CUT&Tag
Omni-ATAC-seq
DMSO, BG4 CUT&Tag*
PDS, BG4 CUT&Tag*
BG4 CUT&Tag#
iMab CUT&Tag
BG4 CUT&Tag^
ATAC-seq
PDS Chem-map
SG4 CUT&Tag
HEK293T cells
Count
Correlation
0.2
0.4
0.6
0.8
1.0
0
5
15
25

Supplementary Figure 4. Untargeted CUT&Tag datasets from HEK293T cells correlate with genome-wide maps of G-quadruplex (G4) presence and chromatin accessibility. (A) Replicate-averaged counts-per-million reads (CPM)-normalized read counts of HEK293T cell-derived libraries and called peaks at a representative locus on chromosome 12. Libraries (Supplementary Table 10) were plotted on the indicated axes, with samples matched to respective controls. BG4 CUT&Tag libraries from different sources are distinguished with superscripts ([*]ref. (18), [^]ref. (29), [#]ref. (30)) as in Supplementary Table 9. Tn5-derived libraries (ATAC-seq, Omni-ATAC-seq, CUT&Tag, and Chem-map) were normalized for library complexity by downsampling, and all libraries were deduplicated prior to read count averaging across replicates and peak calling. **(B)** Clustered heatmap of Pearson's correlation coefficients of normalized read counts from the above libraries across any site of signal enrichment of each sample. The correlation value distribution histogram is plotted within the Pearson's correlation coefficient color scale (top left). Tn5-derived negative control datasets are indicated by shaded rectangles.

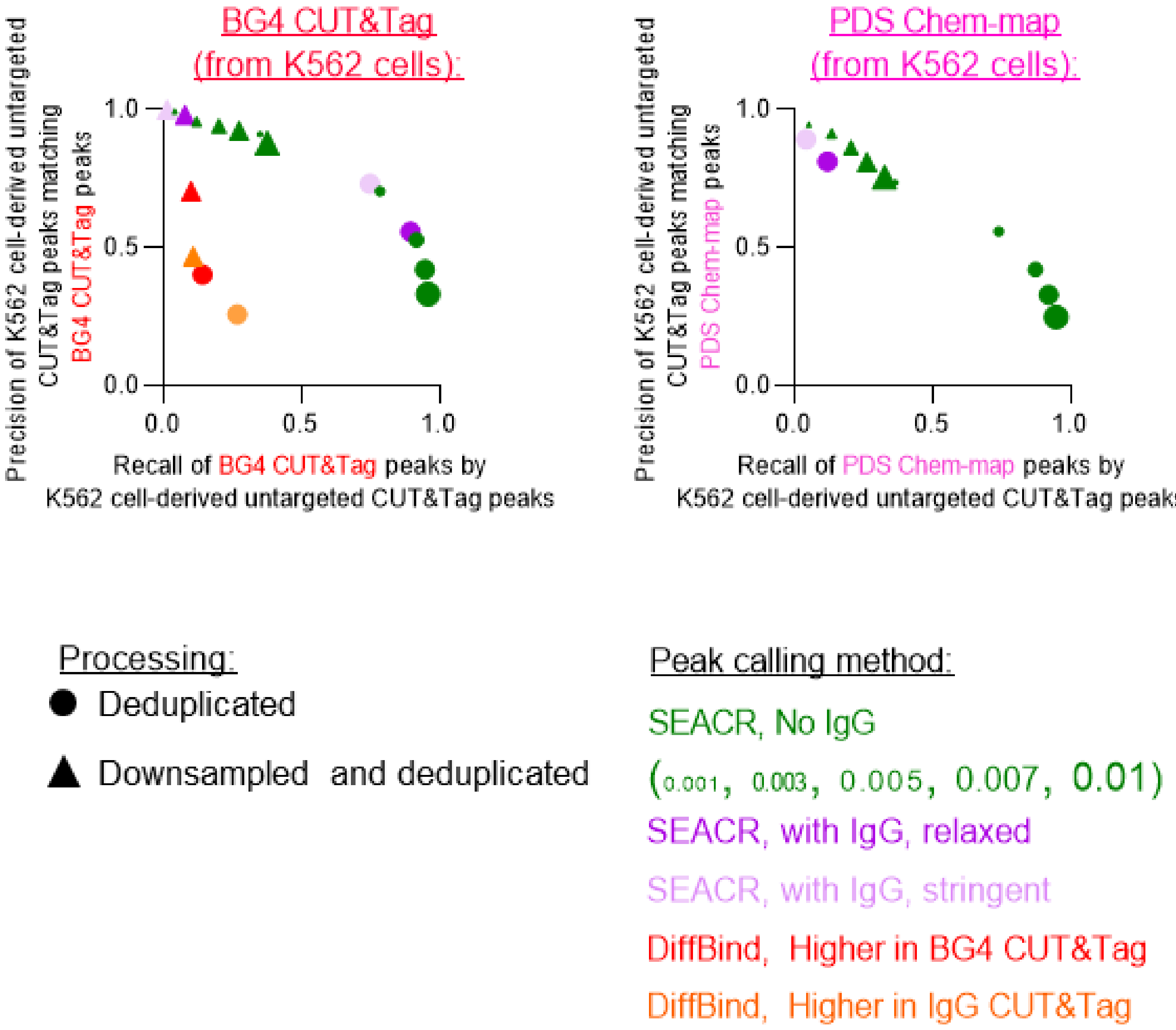

Supplementary Figure 5. Use of matched negative control datasets when calling peaks in Tn5-derived G4-mapping libraries limits recall of G4s mapped by BG4 ChIP-seq. Precision-Recall plots of replicate-conserved peaks called from BG4 CUT&Tag (n=3) and PDS Chem-map (n=2) libraries when compared to a reference BG4 ChIP-seq library (n=3), all in K562 cells.

SUPPLEMENTARY REFERENCES

1. Edgar,R., Domrachev,M. and Lash,A.E. (2002) Gene Expression Omnibus: NCBI gene expression and hybridization array data repository. *Nucleic Acids Res.*, **30**, 207–210.

2. Hu,C.-L., Chen,B.-Y., Li,Z., Yang,T., Xu,C.-H., Yang,R., Yu,P.-C., Zhao,J., Liu,T., Liu,N., *et al.* (2022) Targeting UHRF1-SAP30-MXD4 axis for leukemia initiating cell eradication in myeloid leukemia. *Cell Res.*, **32**, 1105–1123.

3. Hsieh,E., Janssens,D.H., Paddison,P.J., Browne,E.P., Henikoff,S., OhAinle,M. and Emerman,M. (2023) A modular CRISPR screen identifies individual and combination pathways contributing to HIV-1 latency. *PLOS Pathog.*, **19**, e1011101.

4. Ma,J., Zhou,Y., Pan,P., Yu,H., Wang,Z., Li,L.L., Wang,B., Yan,Y., Pan,Y., Ye,Q., *et al.* (2023) TRABID overexpression enables synthetic lethality to PARP inhibitor via prolonging 53BP1 retention at double-strand breaks. *Nat. Commun.*, **14**, 1810.

5. Zhang,X., Ge,L., Jin,G., Liu,Y., Yu,Q., Chen,W., Chen,L., Dong,T., Miyagishima,K.J., Shen,J., *et al.* (2024) Cold-induced FOXO1 nuclear transport aids cold survival and tissue storage. *Nat. Commun.*, **15**, 2859.

6. Li,C., Yin,Z., Xiao,R., Huang,B., Cui,Y., Wang,H., Xiang,Y., Wang,L., Lei,L., Ye,J., *et al.* (2022) G-quadruplexes sense natural porphyrin metabolites for regulation of gene transcription and chromatin landscapes. *Genome Biol.*, **23**, 259.

7. Kaya-Okur,H.S., Wu,S.J., Codomo,C.A., Pledger,E.S., Bryson,T.D., Henikoff,J.G., Ahmad,K. and Henikoff,S. (2019) CUT&Tag for efficient epigenomic profiling of small samples and single cells. *Nat. Commun.*, **10**, 1930.

8. Fu,S., Wang,Y., Bin,E., Huang,H., Wang,F. and Tang,N. (2023) c-JUN-mediated transcriptional responses in lymphatic endothelial cells are required for lung fluid clearance at birth. *Proc. Natl. Acad. Sci.*, **120**, e2215449120.

9. Chen,X., Li,Y., Zhu,F., Xu,X., Estrella,B., Pazos,M.A., McGuire,J.T., Karagiannis,D., Sahu,V., Mustafokulov,M., *et al.* (2023) Context-defined cancer co-dependency mapping identifies a functional interplay between PRC2 and MLL-MEN1 complex in lymphoma. *Nat. Commun.*, **14**, 4259.

10. Guo,T., Hu,S., Xu,W., Zhou,J., Chen,F., Gao,T., Qu,W., Chen,F., Lv,Z. and Lu,L. (2023) Elevated expression of histone deacetylase HDAC8 suppresses arginine-proline metabolism in necrotizing enterocolitis. *iScience*, **26**, 106882.

11. Zhang,D., Zhu,Y., Ju,Y., Zhang,H., Zou,X., She,S., Zhu,D. and Guan,Y. (2023) TEAD4 antagonizes cellular senescence by remodeling chromatin accessibility at enhancer regions. *Cell. Mol. Life Sci.*, **80**, 330.

12. Wang,F., Gao,Y., Xue,S., Zhao,L., Jiang,H., Zhang,T., Li,Y., Zhao,C., Wu,F., Siqin,T., *et al.* (2023) SCARB2 drives hepatocellular carcinoma tumor initiating cells via enhanced MYC transcriptional activity. *Nat. Commun.*, **14**, 5917.

13. Jiang,H., Bian,W., Sui,Y., Li,H., Zhao,H., Wang,W. and Li,X. (2022) FBXO42 facilitates Notch signaling activation and global chromatin relaxation by promoting K63-linked polyubiquitination of RBPJ. *Sci. Adv.*, **8**, eabq4831.

14. Tubío-Santamaría,N., Jayavelu,A.K., Schnoeder,T.M., Eifert,T., Hsu,C.-J., Perner,F., Zhang,Q., Wenge,D.V., Hansen,F.M., Kirkpatrick,J.M., *et al.* (2023) Immunoproteasome function maintains oncogenic gene expression in KMT2A-complex driven leukemia. *Mol. Cancer*, **22**, 196.

15. Li,Y., Goldberg,E.M., Chen,X., Xu,X., McGuire,J.T., Leuzzi,G., Karagiannis,D., Tate,T., Farhangdoost,N., Horth,C., *et al.* (2022) Histone methylation antagonism drives tumor immune evasion in squamous cell carcinomas. *Mol. Cell*, **82**, 3901-3918.e7.

16. Ramasamy,S., Aljahani,A., Karpinska,M.A., Cao,T.B.N., Velychko,T., Cruz,J.N., Lidschreiber,M. and Oudelaar,A.M. (2023) The Mediator complex regulates enhancer-promoter interactions. *Nat. Struct. Mol. Biol.*, **30**, 991–1000.

17. Hui,W.W.I., Simeone,A., Zyner,K.G., Tannahill,D. and Balasubramanian,S. (2021) Single-cell mapping of DNA G-quadruplex structures in human cancer cells. *Sci. Rep.*, **11**, 23641.

18. Li,C., Wang,H., Yin,Z., Fang,P., Xiao,R., Xiang,Y., Wang,W., Li,Q., Huang,B., Huang,J., *et al.* (2021) Ligand-induced native G-quadruplex stabilization impairs transcription initiation. *Genome Res.*, **31**, 1546–1560.

19. Gail,E.H., Healy,E., Flanigan,S.F., Jones,N., Ng,X.H., Uckelmann,M., Levina,V., Zhang,Q. and Davidovich,C. (2024) Inseparable RNA binding and chromatin modification activities of a nucleosome-interacting surface in EZH2. *Nat. Genet.*, **56**, 1193–1202.

20. Heidari,N., Phanstiel,D.H., He,C., Grubert,F., Jahanbani,F., Kasowski,M., Zhang,M.Q. and Snyder,M.P. (2014) Genome-wide map of regulatory interactions in the human genome. *Genome Res.*, **24**, 1905–1917.

21. Shen,J., Varshney,D., Simeone,A., Zhang,X., Adhikari,S., Tannahill,D. and Balasubramanian,S. (2021) Promoter G-quadruplex folding precedes transcription and is controlled by chromatin. *Genome Biol.*, **22**, 143.

22. Esnault,C., Magat,T., Zine El Aabidine,A., Garcia-Oliver,E., Cucchiarini,A., Bouchouika,S., Lleres,D., Goerke,L., Luo,Y., Verga,D., *et al.* (2023) G4access identifies G-quadruplexes and their associations with open chromatin and imprinting control regions. *Nat. Genet.*, **55**, 1359–1369.

23. Yu,Z., Spiegel,J., Melidis,L., Hui,W.W.I., Zhang,X., Radzevičius,A. and Balasubramanian,S. (2023) Chem-map profiles drug binding to chromatin in cells. *Nat. Biotechnol.*, **41**, 1265–1271.

24. Galli,S., Melidis,L., Flynn,S.M., Varshney,D., Simeone,A., Spiegel,J., Madden,S.K., Tannahill,D. and Balasubramanian,S. (2022) DNA G-Quadruplex Recognition In Vitro and in Live Cells by a Structure-Specific Nanobody. *J. Am. Chem. Soc.*, **144**, 23096–23103.

25. Hon,J., Martínek,T., Zendulka,J. and Lexa,M. (2017) pqsfinder: an exhaustive and imperfection-tolerant search tool for potential quadruplex-forming sequences in R. *Bioinformatics*, **33**, 3373–3379.

26. Kent,W.J., Sugnet,C.W., Furey,T.S., Roskin,K.M., Pringle,T.H., Zahler,A.M. and Haussler, and D. (2002) The Human Genome Browser at UCSC. *Genome Res.*, **12**, 996–1006.

27. Nassar,L.R., Barber,G.P., Benet-Pagès,A., Casper,J., Clawson,H., Diekhans,M., Fischer,C., Gonzalez,J.N., Hinrichs,A.S., Lee,B.T., *et al.* (2023) The UCSC Genome Browser database: 2023 update. *Nucleic Acids Res.*, **51**, D1188–D1195.

28. Marsico,G., Chambers,V.S., Sahakyan,A.B., McCauley,P., Boutell,J.M., Antonio,M.D. and Balasubramanian,S. (2019) Whole genome experimental maps of DNA G-quadruplexes in multiple species. *Nucleic Acids Res.*, **47**, 3862–3874.

29. Esain-Garcia,I., Kirchner,A., Melidis,L., Tavares,R. de C.A., Dhir,S., Simeone,A., Yu,Z., Madden,S.K., Hermann,R., Tannahill,D., *et al.* (2024) G-quadruplex DNA structure is a positive regulator of MYC transcription. *Proc. Natl. Acad. Sci.*, **121**, e2320240121.

30. Zanin,I., Ruggiero,E., Nicoletto,G., Lago,S., Maurizio,I., Gallina,I. and Richter,S.N. (2023) Genome-wide mapping of i-motifs reveals their association with transcription regulation in live human cells. *Nucleic Acids Res.*, **51**, 8309–8321.

31. Aldana,J., Gardner,M.L. and Freitas,M.A. (2023) Integrative Multi-Omics Analysis of Oncogenic EZH2 Mutants: From Epigenetic Reprogramming to Molecular Signatures. *Int. J. Mol. Sci.*, **24**, 11378.

32. Zheng,K., Zhang,J., He,Y., Gong,J., Wen,C., Chen,J., Hao,Y., Zhao,Y. and Tan,Z. (2020) Detection of genomic G-quadruplexes in living cells using a small artificial protein. *Nucleic Acids Res.*, **48**, 11706–11720.

33. Love,M.I., Huber,W. and Anders,S. (2014) Moderated estimation of fold change and dispersion for RNA-seq data with DESeq2. *Genome Biol.*, **15**, 550.

34. Stark,R. (2011) DiffBind: differential binding analysis of ChIP-Seq peak data. *Bioconductor*, **v3.20**.
